# Behçet’s Disease In Children And Adults Of Sub-Saharan Ancestry: A Systematic Review And Meta-Analysis

**DOI:** 10.1007/s12016-025-09085-8

**Published:** 2025-08-14

**Authors:** Benoit Suzon, Arthur Felix, Fabienne Louis-Sidney, Esther Dalmasie, Sunniva Donat, Christophe Deligny, Aurore Abel, Eleonore de Fritsch

**Affiliations:** 1 Department of Internal Medicine, University Hospital of Martinique, Martinique, France; 2 Department of General Pediatrics, Competence Centre for Rare Pediatric Inflammatory Rheumatisms and Systemic Autoimmune Diseases (RAISE) Antilles-Guyane, University Hospital of Martinique, Martinique, France; 3 EpiCliV Research Unit, University of the French West-Indies, Martinique, France; 4 Department of Rheumatology, University Hospital of Martinique, Martinique, France; 5 Department of Research and Clinical Innovation, University Hospital of Martinique, Martinique, France

**Keywords:** Behçet’s disease, Sub-Saharan ancestry, Ethnicity, Systematic review, Meta-analysis, Black

## Abstract

**Background:**

The characteristics of Behçet’s disease (BD) in individuals of Sub-Saharan ancestry (sub-SA) are poorly understood.

**Methods:**

Herein, we conducted a PRISMA-compliant systematic review using the PubMed/Medline, Scopus, and Web of Science databases. Articles published up to September 1, 2023 were searched with the following keywords: "Behçet's disease" OR "Behcet's syndrome" AND "sub-Saharan African" OR "Black" OR “African”. Data on the year, type and country of study, sample size, region of origin, nationality, age, sex, time to diagnosis, death, HLAB51 status, mucosal, and organ involvement were collected. Involvement of the central nervous system was retained on the basis of objective criteria, and dichotomized into parenchymal or non-parenchymal/vascular lesions. The pooled frequency of patients’ main characteristics was calculated using a DerSimonian-Laird random-effects meta-analysis.

**Results:**

This study included 42 full-text reports, with study periods ranging from 1970 to 2023. Overall, 230 adult patients (69% of males) were included, of whom 195 (85%) were from sub-Saharan African countries, 22 (10%) patients were from the Caribbean, and 13 (5%) patients were from the Americas, including 12 African Americans, and 1 African Brazilian. Oral and genital ulcers were reported in 98% [95% CI 91 to 100%] and 85% [72 to 92%], respectively. Ocular involvement occurred in 43% [31 to 56%] of patients. Central nervous system (CNS) involvement affected 39% [25 to 54] of the patients. Among them, 30% of patients had a cerebro-vascular disease, and 72% had a parenchymal involvement. The patients were mostly treated with oral steroids and colchicine, and remission was achieved in 35/54 (65%) patients, but 15 (69%) of them suffered severe sequelae, particularly ophthalmological and neurological.

**Conclusion:**

Behçet’s disease in patients of sub-Saharan ancestry appears to be predominantly HLA B51/B5-negative, and more severe than in other ethnicities, owing to a high prevalence of CNS involvement.

**Supplementary Information:**

The online version contains supplementary material available at 10.1007/s12016-025-09085-8.

## Introduction

Behçet’s disease (BD) is a chronic multisystem inflammatory disorder characterized by the development of recurrent oral and genital aphthous ulcers, skin lesions, and uveitis [1]. BD also presents with other symptoms, with manifestations involving the gastrointestinal tract, blood vessels of variable size, or central nervous system (CNS) ranging from mild to life-threatening [1].

The global pooled BD prevalence is estimated 10.3 [95%CI 6.1 to 17.7] [1]; however, this incidence shows regional disparity, with higher rates in countries located along the ancient Silk Road, affecting 80 to 600 per 100,000 people in Turkey [2–4] and 13.5 to 80 per 100,000 people in Japan, Iran, and Saudi Arabia [5–7]. Incidence estimates per 100,000 people are much lower in North American (0.3 to 5.2 in the USA [8]) and European (0.3 to 2.3 in Germany [9], 0.6 in England [10], 1.2 to 4.9 in Sweden [11], 1.5 in Portugal [12], 7.1 in France [13], and 15.9 in southern Italy) countries [14].

The incidence of BD is strongly influenced by ethnicity, as shown in studies conducted among non-Caucasian inhabitants of European countries. Specifically, the BD prevalence is higher in individuals from high-prevalence regions (i.e., North Africa) than in the Caucasian population, regardless of age at the time of immigration [13]. Genetic determinants have also been identified [15], such as HLA-B51, which is present in 40 to 70% of patients with BD from the Middle East and Asia and 13% of patients with BD from Europe and North America [16], and is known to increase the risk of BD occurrence among carriers six-fold [17].

Early recognition of BD is important given its high rates of morbidity and mortality. Male sex, CNS involvement, and vascular involvement [1] have all been associated with the worst disease prognosis.

With regard to paediatric BD, it is estimated to affect 5–15.5% of the total BD population. According to a study conducted at a reference centre in China, the frequency of manifestations may vary between children and adults (for example, arthralgia and panuvitis are less common in children than in adults) [1, 18, 19].

The most robust data on BD has been found in the Arabic, Asian, North African, and Caucasian populations [1]. Conversely, data on BD in individuals of sub-Saharan ancestry (sub-SA) is scarce. The largest study conducted amongst individuals of sub-SA only included 50 patients, but suggested a higher frequency of CNS involvement in individuals of sub-SA than other ethnicities [20].

Individuals of sub-SA constitute a growing and predominantly young population with a worldwide distribution (e.g., mostly sub-Saharan countries, Europe, Caribbean, the Americas), in which immune-mediated diseases (e.g. systemic lupus erythematosus) tend to start earlier than in other ethnicities. Whether this younger age at diagnosis is also observed in BD remains to be investigated; however, the only available comparative study conducted in one hospital did not find differences regarding age at diagnosis [20]. As such, whether this age at diagnosis in patients of sub-SA is consistent in other sub-SA populations is unknown, as are distribution of BD manifestations and outcomes [1].

Overall, despite the large and worldwide distributed population of individuals of this ethnicity, little is known about BD in patients of sub-SA, and a better characterization is therefore needed. In this context, we elected to conduct a systematic review to uncover the international evidence of BD in patients of sub-SA, and to identify areas for future research in BD, as well as in patients of sub-SA. As very little is known on the subject, the objectives of this systematic review were to determine the epidemiology of BD in pediatric and adult individuals of sub-SA.

## Methods

### Design

This review adhered to the 2020 Preferred Reporting Items for Systematic Reviews and Meta-Analyses (PRISMA) guidelines [21]. The review protocol has been registered in the International Prospective Register of Systematic Reviews (PROSPERO), under the registration number CRD42023478282.

### Data Sources and Search Strategies

Two independent authors (AF and EDF) searched the PubMed, Embase, Scopus, and Web of Science databases for studies published from database inception to September 1, 2023. On November 29, 2024, relevant studies published since September 1, 2003, were further searched. The keyword combinations used were “Behcet’s syndrome [MeSH]” OR “Behcet’s disease” AND “Black people [MeSH]” OR Sub-Saharan African People [MeSH] OR “Africa”. The authors also searched databases dedicated to publications from the African continent, namely African Journals Online and Africa Index Medicus, using "Behçet's disease" OR "Behçet's syndrome" (Table S1 and S2). Relevant articles could be added manually if they were absent from database search results; grey literature search was not performed.

### Study Selection

Following the elimination of duplicates, two authors (AF, EDF) were independently involved in the study selection and data extraction. Any study design was included. The included population comprised patients of sub-SA with a diagnosis of BD according to authors, regardless of their country of residence. No intervention or comparison was mandatory, and the variables assessed included any information available about BD in patients of sub-SA. No filters were applied for language or study design. We decided to also include case series and case reports to provide the most exhaustive possible analysis of BD in this population group given the anticipated paucity of BD data in this population group.

Secondly, we eliminated irrelevant publications based on titles and abstracts, and subsequently further excluded irrelevant publications based on the full text. In cases of disagreement between experts, a third expert (BS) was consulted to make the final decision. If the ethnicity of the study population was not clearly stated in the title or abstract, the full text was reviewed prior to inclusion or exclusion. If the full text was not available, the first and/or corresponding authors were contacted to obtain the full text. Reports were excluded if they did not mention Behçet's disease in a patient of sub-SA, if the data on Behçet's disease were insufficient, or if they included only the patient’s medical history, without additional information.

### Data Extraction

We collected all available variables about BD in patients of sub-SA, including the year of study publication; study type; country where the study was conducted; sample size; participants’ ethnicity, region of origin, nationality, age, sex, and time to diagnosis or death. Further, information was collected about BD symptoms, including fever; mucosal and skin lesions (genital, oral, and skin ulcers, erythema nodosum-like lesions, pseudofolliculitis, pathergy test-related pustule, acneiform rash), ocular involvement; arthritis; vascular manifestations (deep and superficial venous thrombosis, aneurysm, occlusion); thoracic (pulmonary embolism, dyspnea, hemoptysis, pleuresia, pericardial effusion), renal (hematuria, renal insufficiency) or gastrointestinal (ascites, diarrhea, abdominal pain, intestinal hemorrhage) involvement; and treatments.

The “neurological involvement” variable was dichotomized into central and peripheral nervous system involvement. CNS involvement was classified based on objective arguments and dichotomized into parenchymal and nonparenchymal/vascular lesions. The presence or absence of headaches was also recorded, but isolated headaches were not considered as a marker of CNS involvement. We further extracted any outcome reported by studies. Organ involvement was considered to be absent in case reports, case series, or prospective studies if it was not mentioned by the authors. However, if organ involvement was not mentioned in retrospective studies, we considered it as potentially missing data.

Two reviewers assessed the study quality and risk of bias (Supplementary Table 3) using the NIHLBI study quality assessment tools [22], with quality classified as ranging from “poor” to “good” (Supplementary Data) for observational and cross-sectional studies. For case series and case reports, the quality was assessed by 4 reviewers using the and the Joanna Briggs Institute checklist for case reports [23, 24].

### Data Analysis and Interpretation

We calculated the ISG [25] and ICBD [26] score criteria for each adult patient, and classified them as having BD when the major criterion (recurrent oral ulceration) and two minor criteria (among recurrent genital ulceration, eye lesions, skin lesions or positive pathergy test) of the ISG were noted, or when 4 points in the ICBD criteria were met. ICBD criteria assigned 2 points for oral aphthosis, genital aphthosis, and ocular lesion, and 1 point for skin lesion, neurological manifestations, vascular manifestation or positive pathergy test. The classification criteria set of the Peadiatric Behçet’s Disease (PEBD) study group was applied for pediatric patients [27]. Quantitative variables are described as medians and interquartile ranges. Qualitative variables are described as numbers with percentages. The pooled frequency of patients’ main characteristics was calculated using a DerSimonian-Laird random-effects meta-analysis, and only random-effects meta-analysis was retained considering heterogeneity between the types of included study reports. To assess the heterogeneity across studies in the meta-analysis, the I^2^-statistic was calculated. The heterogeneity was classified as low if I^2^ value was 25% or less; moderate if I^2^ value was 50% as moderate; and high if I^2^ value was 75% or more [28]. The statistical significance of results was retained if the p-value was lower than 0.05. The meta-analysis was performed using the “Metaprop” function in the R package “meta”, release 7.0–0 (2024–01-11), R Foundation for Statistical Computing, Vienna, Austria. In addition to the meta-analysis, the authors also conducted a systematic review without meta-analysis for the data relating to the pediatric population, in the form of a narrative summary of the data given the limited number of cases. No ethical approval or patient consent was required.

## Results

**Fig. 1:**
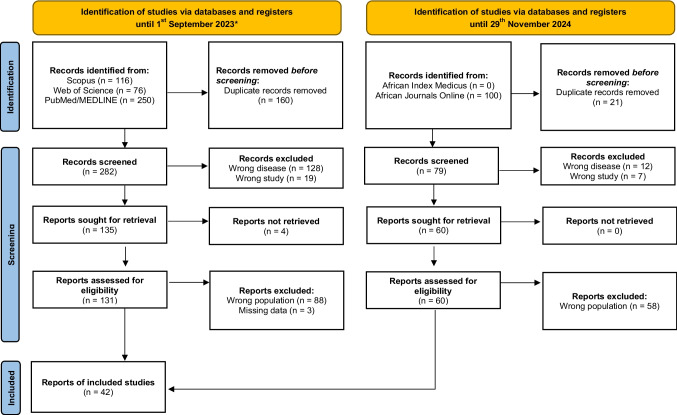
Study flow diagram according to PRISMA guidelines of the selection and inclusion of articles in the systematic literature review on Behcet’s disease in children and adults of sub-Saharan ancestry. * Literature analysis was performed again on the 29.^th^ November 2024, but identified no further relevant articles (n = 6 new articles found)

Overall, we identified 542 articles from the databases, of which 40 full-text reports on adults of sub-SA with BD [20, 29–66] and two articles on children [67, 68] were finally included, with the publication period of the studies ranging from 1970 to 2023 (Fig. 1).

### General Characteristics of Included Primary Studies

The study designs were as follows: case reports or series, 35 articles (57 adults and 2 children) [30–33, 35, 37–41, 43–48, 50–58, 60–68]; six cross-sectional retrospective studies articles (158 patients) [20, 34, 36, 42, 49, 59]; including a comparative analysis (50 sub-Saharan patients among 158 patients) [59]; and one cross-sectional prospective study (15 patients) [29]. Studies were monocentric, except for one retrospective multicenter study conducted from 1970 to 1995 in Senegal [34].

### I. Pediatric Patients

Two pediatric cases (one male and one female) with BD were retrieved, both from North America (USA).

The girl was 13 years old (sociodemographic and economic characteristics were unknown) fulfilled the PEBD classification criteria for pediatric BD, ICBD criteria, but did not correspond to the ISG criteria owing to the oral and genital ulcerations without other minor criterion in this last set. The patient had no family history of BD, and was admitted and treated for complete occlusion of the left popliteal artery revealed by leg pain, pulmonary aneurysms revealed by hemoptysis and hypoxia; intracardiac thrombi. After anticoagulation therapy (enoxaparin 1 mg/kg per day, discontinued after two hospitalizations, with no specific time frame, due to recurrent minor hemoptysis) and five infusions of cyclophosphamide (750 to 1000 mg/m^2^), maintenance therapy consisted of combination of oral steroids (1 mg/kg per day) with infliximab (5 mg/kg every 8 weeks), and the authors reported no further events since the introduction of this treatment, the last episode of hemoptysis occurring 10 months earlier [67].

The second child was a 10-year-old boy with no family history of BD (sociodemographic and economic characteristics were unknown) who did not fulfill the ISG criteria set for definite BD, had less than 4 points in the ICBD criteria set, and did not fulfill the PEBD classification criteria for pediatric BD. He had no actual or medical history of oral or genital ulcerations, no family history of BD, and presented solely with hemoptysis and bilateral lower lobe fusiform pulmonary aneurysms. Histological analysis on a post-mortem lung sample revealed a necrotizing lymphocytic vasculitis without granuloma in the pulmonary arteries of both lower lobes and their major branches. The patient died 11 days after the onset of hemoptysis despite a vascular embolization procedure performed 3 days after admission, which stopped the bleeding. No specific context or cause of death was given [68].

These two pediatric cases in sub-SA patients are the only ones found during this systematic review of the literature. The first child presented positive anti-nuclear, double-stranded DNA, and anti-phospholipid antibodies, meeting the 2019 ACR/EULAR criteria for systemic lupus erythematosus [69], more or less associated with an anti-phospholipid syndrome. It is possible that the diagnosis of BD was made in view of the genital ulcerations, which are rare in systemic lupus but are major manifestations in BD. Another argument for the diagnosis of BD is the pulmonary aneurysms and the control of the disease after treatment with prednisone and anti-TNF alpha [1].

The diagnostic arguments are more limited in the second patient, since he had no oral or genital ulceration. An ADA2 deficiency could have explained the vascular aneurysms and necrotizing vasculitis on histology, but its isolated pulmonary arterial presentation without skin lesions or neurological manifestations seems unlikely [70]. Also, familial history was absent. However, genetic analyses for this diagnosis were not performed, nor was research into HLA B51 for BD. However, apart from BD, no other obvious diagnosis explains the pulmonary aneurysms and the histology of necrotizing vasculitis without granuloma. The outcome in this patient was unfavorable, possibly related to the progression of vascular lesions in a patient who was not diagnosed with BD at the time and was not treated with immunosuppressants.

The isolated involvement of the pulmonary arteries made diagnosis difficult, but it should be noted that the pediatric BD cases identified in this review represent less than 1% of the total number of cases, which is lower than the rates usually observed (4 to 26%) [71]. This may be the result of a lower incidence/prevalence of BD in children with sub-SA, but also of a lack of knowledge of the disease in this population and a lack of diagnosis, possibly due to difficult access to care.

### II. Adult Patients

Overall, 230 adult patients (69% [CI95%; 63 to 75%] male, figure S2) were included in this study. The forest plots of grouped frequencies of clinical and biological characteristics are presented in Fig. 4 to 12, as well as in a summary in Table 1 and Fig. 2.

**Table 1:** Behcet’s disease in adult patients of sub-Saharan ancestry: summary of characteristics by study

	**Case reports or case-series**	**Ndiaye et al. **[20]	**Savey et al.** [59]	**Lannuzel et al.** [42]	**Niang et al.** [49]	**Gaye et al.** [36]	**Dia et al.** [34]	**Ajose et al.** [29]	**Total** n (%) or (% [95%CI]) ^**#**^
Study period	1994 to 2023	2000 to 2013	1974 to 2010	1989 to 1999	1997 to 2006	2012 to 2018	1970 to 1995	2007 to 2011	1994 to 2023
Country / Regions	Various	Senegal / Africa	France / Europe	Guadeloupe / Caribbean	Senegal / Africa	Senegal / Africa	Senegal / Africa	Nigeria / Africa	Various
**General characteristics**									
Number of patients	57 (100)	50 (100)	50 (100)	13 (100)	12 (100)	16 (100)	17 (100)	15 (100)	230 (100)
Male (n, %)	41 (71)	31 (62)	39 (78)	8 (62)	8 (67)	14 (88)	12 (71)	9 (60)	69% [63 to75]
Age, years	31 [26 to 38], median value	32 [18 to 67], mean value	32 [25 to 41], median value	N/A	24 [18 to 54], mean value	40 [18 to 71] * mean value	28 [11 to 42], mean value	27 [16 to 40], mean value	31 [26 to 37]
**Clinical and biological data**									
Oral ulcers	52 (91)	50 (100)	50 (100)	12 (92)	12 (100)	16 (100)	16 (94)	15 (100)	98% [91 to 100]
Genital ulcers	42 (76)	49 (98)	31 (62)	9 (69)	11 (92)	14 (88)	14 (82)	14 (93)	85% [72 to 92]
Skin involvement	32 (56)	N/A	N/A	10 (77)	6 (50)	2 (13)	N/A	13 (87)	57% [31 to 80]
Joint involvement	27 (47)	20 (40)	23 (46)	10 (77)	6 (50)	1 (6)	8 (47)	12 (80)	48% [33 to 63]
Gastrointestinal involvement	9 (16)	6 (12)	N/A	1 (8)	2 (17)	N/A	2 (12)	1 (7)	13% [9 to 19]
Vascular involvement	18 (33)	9 (18)	27 (54)	3 (23)	0 (0)	1 (6)	6 (35)	3 (20)	23% [13 to 37]
Ocular involvement	23 (40)	22 (44)	26 (53)	8 (62)	8 (67)	2 (12)	10 (59)	12 (80)	43% [31 to 56]
Central nervous system involvement	22 (38)	12 (24)	24 (48)	6 (54)	N/A	13 (81)	5 (29)	2 (13)	39% [25 to 54]
Fever	13 (24)	0	N/A	3 (23)	N/A	9 (56)	N/A	0	8% [1 to 47]
HLA-B51	2/34 (6)	N/A	10/34 (29)	0/6 (0)	N/A	N/A	0/2 (0)	N/A	10% [2 to 35]

**Fig. 2:**
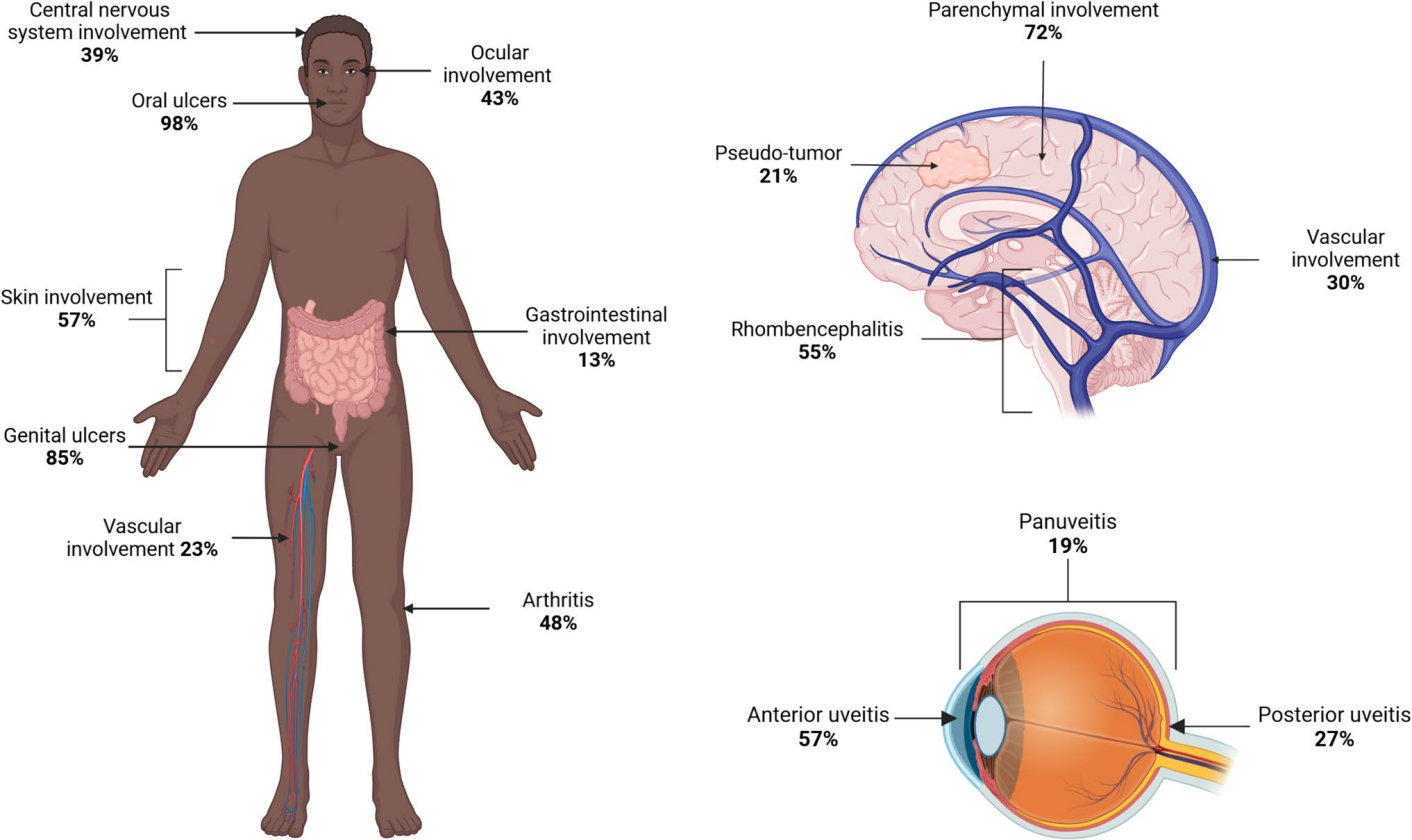
Summary of the distribution of Behcet’s disease manifestations in adult individuals of sub-Saharan ancestry. NB: Detailed percentages of neurological and ocular damage are given for patients affected with these condition. The illustration has been created with Biorender.com®

N/A: data not available; * result must be interpreted with caution because it is mostly the age at the diagnosis of neurological Behçet syndrome (number of positive results/n test performed, %); proportion is related to the total number of patients, unless specified otherwise. ^#^ Details on heterogeneity and metanalysis are given in Fig. 2

Four patients had a familial history of BD (2% of all patients in all reports or 4% of patients in reports that clearly mentioned a search for familial history) [20, 43].

One hundred and ninety-five (85%) patients were from sub-Saharan Africa [20, 29, 32, 34, 36–38, 40, 43, 44, 49, 55, 56, 58, 65, 66], but the country was not specified for 52 patients [31, 59, 62]. Twenty two (10%) patients were from the Caribbean [42, 50, 55], but the specific country was not indicated for 4 patients [30, 46, 48, 54]. Finally, 13 (5%) patients were from the Americas (North or South America) [33, 39, 41, 47, 51–53, 57, 61, 63, 64].

**Fig. 3:**
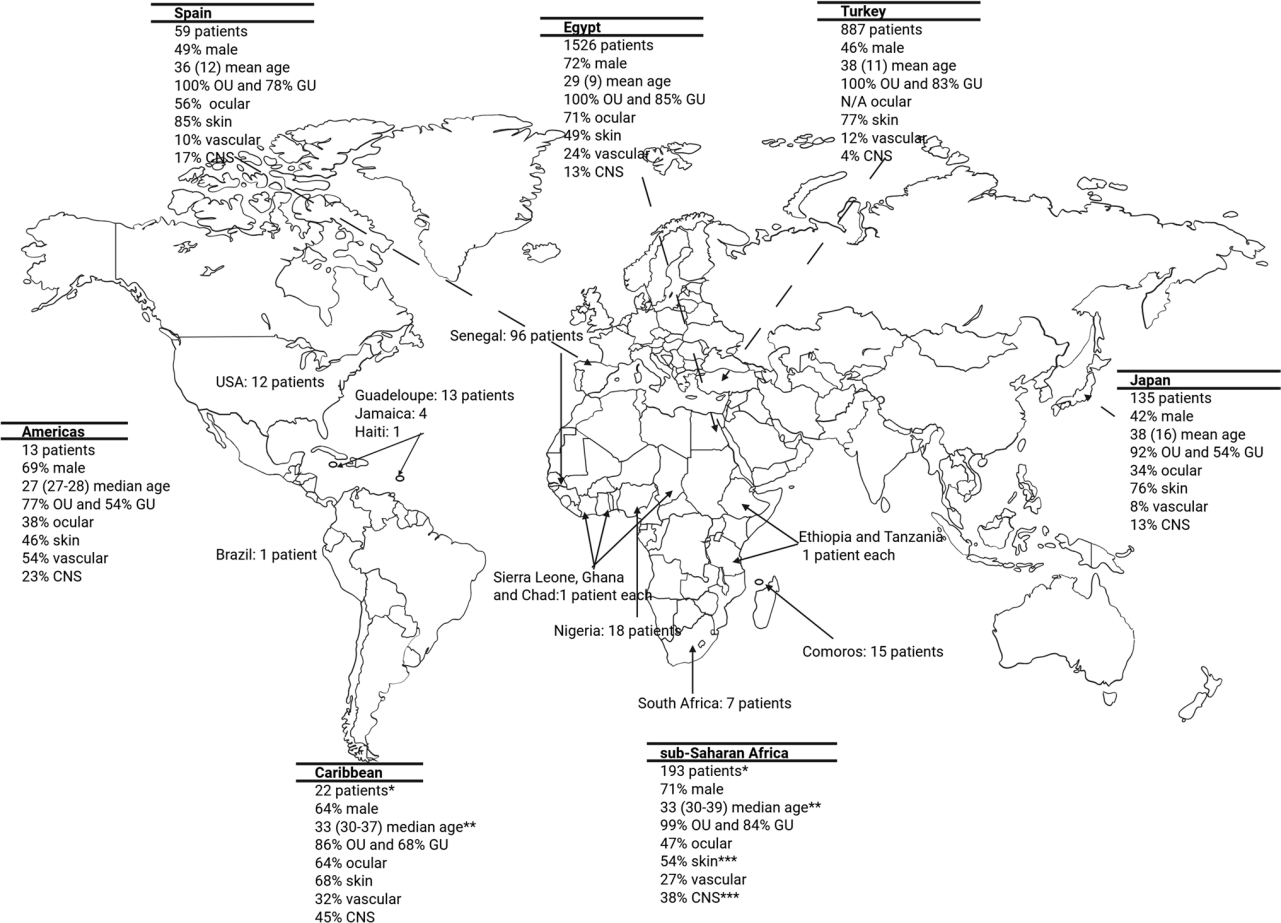
Overview of the geographic distribution of Behçet’s disease (BD) in individuals of sub-Saharan ancestry (sub-SA), showing the number of patients per country and summary of characteristics of patients of sub-SA with BD in the Americas, Caribbean, and sub-Saharan Africa regions. The Americas comprised patients from USA and Brazil. The Caribbean comprised patients from Guadeloupe, Jamaica, and Haiti. The sub-Saharan Africa comprised the other countries. Data of patients from Spain [72] Egypt [73] Turkey [74] and Japan [75] are given for comparative purpose. CNS: central nervous system involvement; GU: genital ulcers; OU: oral ulcers. *The country was unknown for 4 African Caribbean patients and for 52 sub-Saharan African patients. ** data available for 9 African Caribbean patients and for 177 sub-Saharan African patients

The median time to diagnosis was 24 months [17 to 51] in sub-Saharan Africa, 24 months [24 to 27] in the Caribbean, and 9 months [1 to 28] in the Americas. Figure 3 summarize patient characteristics by world regions of origin.

### Prevalence, Classification Criteria Used and Genetic Susceptibility

This analysis included one study reporting the prevalence of BD in individuals of sub-SA [34]. In that study conducted in the unique tertiary care center of the Guadeloupe (French Caribbean island), the BD prevalence was 3 per 100,000 inhabitants between 1989 and 1999 [42].

**Fig. 4:**
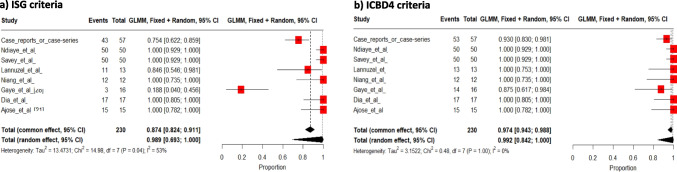
Pooled frequencies of adult individuals of sub-Saharan ancestry with Behçet’s disease meeting ISG (**a**) and ICBD4 criteria (**b**)

Overall, 99% [95% CI 69 to 100] of patients presented with at least one major criterion and two minor criteria of the ISG classification, and 99% [84 to 100%] of patients had a score ≥ 4 when using the ICBD classification (Fig. 4a and 4b). Fever occurred in 8% [1 to 47%] of patients (figure S1), most of whom (7/12, 58%) had neurological Behçet syndrome.

**Fig. 5:**
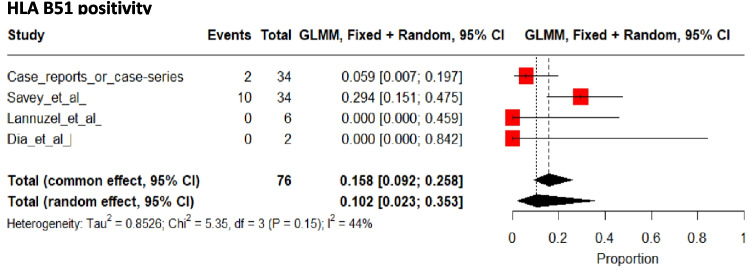
Pooled frequencies of HLA B51 positivity in adult individuals of sub-Saharan ancestry with Behçet’s disease

HLA-B51 was positive in 10% [2 to 35%] of patients (Fig. 5).

### Mucosal and Skin Involvement

**Fig. 6:**
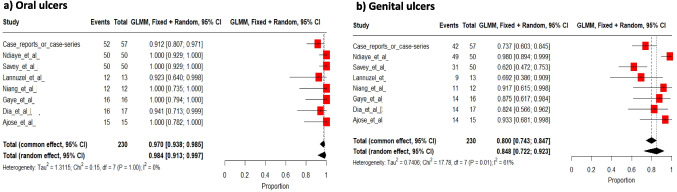
Pooled frequencies of oral (**a**) and genital (**b**) ulcers in adult individuals of sub-Saharan ancestry with Behçet’s disease

Oral and genital ulcers were reported in 98% [91 to 100%] and 85% [72 to 92%] of patients, respectively (Fig. 6a and 6b).

Details of the localization of these oral ulcers are available for 98 patients, showing they were located on the tongue (n = 30/98, 31%), palate (n = 14/98, 14%), or lips (n = 14/98, 14%). One patient had a nasal ulceration.

**Fig. 7:**
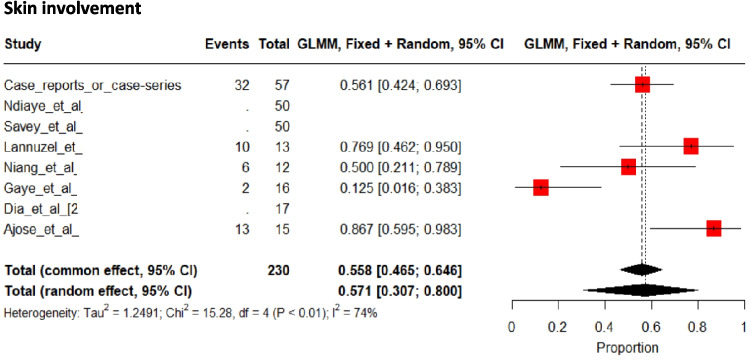
Pooled frequencies of skin involvement related to Behçet’s disease in adult individuals of sub-Saharan ancestry

Global skin involvement was specifically stated in 62 patients (57% [57 to 80%]) (Fig. 7); however, some studies specifically reported skin involvement in patients without reporting global skin involvement in all patients. In this final series of 178 patients, pseudofolliculitis affected 48 (27%) patients, erythema nodosum 34 (19%), sterile pustule eruption 19 (11%), skin ulcers 14 (8%), and acneiform eruption 9 patients (5%). A pathergy test was performed in 140 patients, yielding positive results in 52 (36%).

### Ocular Involvement

No prospective or retrospective study has yet specifically investigated ocular involvement in BD; and only one case series focused on this aspect [55].

**Fig. 8:**
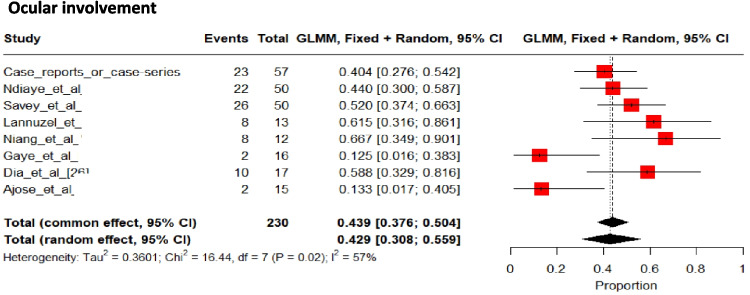
Pooled frequencies of ocular involvement related to Behçet’s disease in adult individuals of sub-Saharan ancestry

Overall, ocular involvement occurred in 43% [95%CI 21 to 56%] (Fig. 8) of patients [20, 29, 35, 36, 38, 42–44, 46, 52–55, 59, 60, 63–66], presenting bilaterally in 74% of cases.

The details regarding ocular involvement were available for 82 patients. Uveitis affected the anterior segment in 57% (47/82) of patients, and 21% of patients had a panuveitis. A hypopyon was prevalent in 4%. Combined posterior uveitis and retinitis affected 22 patients, of which two had retinal detachment, 10 retinal vasculitis, three retinal periphlebitis (including one patient with central retinal vein occlusion), three others retinal infiltrates, two retinal hemorrhages, and two others missing details about retinal involvement. Four patients developed conjunctivitis.

### Neurological Involvement

**Fig. 9:**
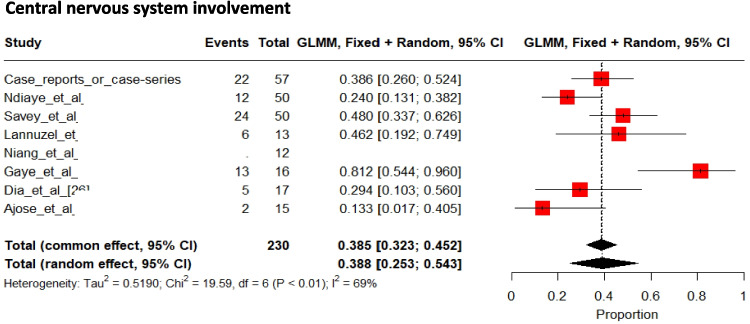
Pooled frequencies of central nervous system involvement related to Behçet’s disease in adult individuals of sub-Saharan ancestry

CNS involvement was reported in 83/218 (39% [95% CI 25 to 54%] (Fig. 9)) patients and peripheral neurological involvement in 5/218 (2%) patients [20, 29, 34, 36–38, 40, 42, 43, 46–48, 50, 53, 55–57, 59–61, 63]. Among the remaining 12 patients (total = 230), 5 were reported to have neurological signs without specific details regarding the definition of neurological involvement [49] and were therefore not included in the following patient description.

Details regarding the neurological involvement were available for 51 patients (47 with central and 4 with peripheral neurological involvement) as two studies reported neurological involvement without details in 37 patients (36 with central and 1 with peripheral involvement) [21, 28, 30, 32, 34, 35, 39, 47–49, 52, 53, 55].

Neurological involvement was the initial symptom of BD in 19 patients, accounting for 48% (19/40) of patients with neurological involvement and 23% (19/84) of patients with or without neurological involvement for whom onset or progression details were available.

### CNS Involvement

#### Parenchymal CNS Involvement

Among 47 patients with CNS involvement, 39 (83%) experienced headache. Motor deficiency or hemiparesis/hemiplegia was reported in 25 (53%) patients, oculomotor nerve palsy in 8 patients, seizure in 7, and facial nerve palsy in 3 patients.

Thirty-four of the 47 patients (72%) had parenchymal involvement [28, 32, 35, 48, 49]. Details were available for 29 patients, among whom rhombencephalitis/brain stem lesions were the most frequent presentation, affecting 55% (16/29) of patients. A pseudotumor was observed in 6 (21%) [36, 40, 43, 56, 57], meningoencephalitis in 7 (24%) [36, 42, 55, 63], and myelitis in 3 (10%) patients, among whom 1 had meningoencephalomyelitis [42]. Four patients had retrobulbar optic neuritis diagnosed on visual evoked potentials [36].

Details of cerebral imaging were available for 31 patients. The brainstem was involved with no further description provided in 14 (45%) patients. The mesencephalon was involved in 7 patients, the diencephalon and pons in 3 patients each, and details were unavailable in 7 patients. The internal capsule was affected in 11 (35%) patients, the thalamus in 9 (29%), the lobar hemispheric regions in 2 (6%), and the periventricular regions in 2 (6%), with 1 (3%) patient having extensive lesions.

Cerebrospinal fluid analysis was performed in 28 patients. Aseptic meningitis was observed in 20 (71%) patients, with a lymphocytic profile noted in 65%, pleocytosis in 10%, and a neutrophilic profile in 5% of patients. Details regarding the other four CSF analyses were lacking. No study reported the CSF levels of interleukin 6.

#### Non-parenchymal CNS Involvement

##### Cerebrovascular and Neuropsychiatric Behçet’s Disease

Fourteen of 47 (30%) patients had involvement of the cerebral arteries or veins (cerebrovascular disease), among whom 10 had cerebral venous thrombosis [36, 37, 42, 43, 47, 48, 50, 55]. One patient presented with both parenchymal and vascular involvement of the CNS. Cerebral venous sinus thrombosis was noted in the superior sagittal sinus in 4 patients, in the lateral sinus in 2 patients, and was multifocal in 2 patients. Finally, four patients had cerebral infarctions [36, 42, 48], and two had cerebral hematomas [36].

Neuropsychiatric manifestations were reported in 20 (43%) patients [29, 34, 46, 50, 63], three with acute psychiatric disorders, 14 with depression, and two with anxiety. Details for one patient were missing.

##### Peripheral Neurological Involvement

Five patients had peripheral nervous system involvement. Data were missing for 2 patients, and the other 3 patients had polyneuropathy [20, 42].

### Extracerebral Vascular Involvement

**Fig. 10:**
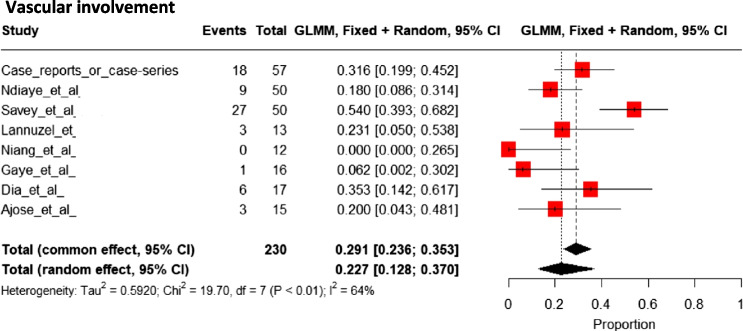
Pooled frequencies of vascular involvement related to Behçet’s disease in adult individuals of sub-Saharan ancestry

Vascular disease was observed in 23% [95%CI 13 to 37%) of patients (Fig. 10) [20, 29, 30, 32, 34, 36, 37, 39–43, 48, 51–53, 55, 57–59, 64], and details were available for 40 patients. Venous involvement was the most frequent, with superficial venous thrombosis in 7 (18%) patients and deep venous thrombosis in 29 (72%) patients. The location of venous thrombosis was variable, with cases including pulmonary artery thrombosis in 4 patients, superior vena cava in 2, intracardiac thrombi in 1, subclavian vein in 2, internal jugular vein in 1, inferior vena cava in 2, renal vein in 1, brachiocephalic vein in 2, sural vein in 1, and upper limb veins without details in one patient.

Arterial occlusion was observed in 7 patients (18%), including occlusion in the internal carotid artery in one patient, the vertebral artery in one patient, the aorta distal to the renal arteries and the superior mesenteric artery in one patient, the distal popliteal artery and the peroneal artery in one patient, and the arteries of the lower limbs in three patients, with no further details.

Aneurysms occurred and were confirmed by angiography in eight patients (12%), with the following distributions noted: pulmonary arteries five patients, thoracic aorta one patient, common carotid artery one patient, vertebral artery in one patient, coronary artery one patient, renal artery one patient, hypogastric artery one patient, axillary artery one patient, internal iliac artery one patient, popliteal artery one patient, superficial femoral artery one patient, and deep femoral artery one patient. Aneurysm rupture was observed in two patients and vertebral artery dissection in one patient.

### Joint Involvement

**Fig. 11:**
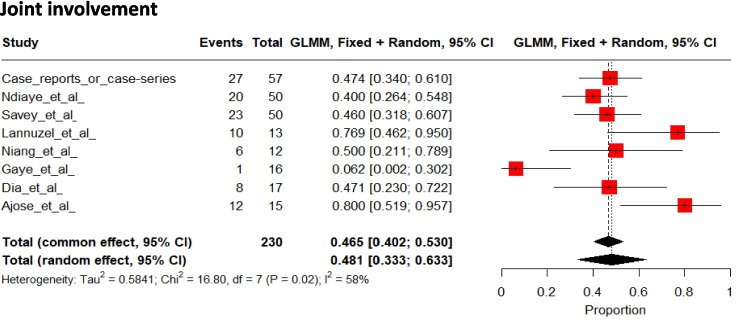
Pooled frequencies of joint involvement related to Behçet’s disease in adult individuals of sub-Saharan ancestry

Joint involvement occurred in 48% [95%CI 33 to 63] of patients (Fig. 11) [20, 29, 31, 33, 34, 36, 38, 42, 43, 46, 48, 49, 55, 58, 59, 62, 63, 65], but many details were missing, and the pattern of joint involvement was presented in only 45 patients. Among these, the oligoarticular pattern was the most frequent, affecting 51% of patients, followed by the polyarticular pattern (40%) and the monoarticular pattern (11%). The knees were the most affected body part, followed by the ankles, wrists, and elbows.

### Gastrointestinal Involvement

**Fig. 12:**
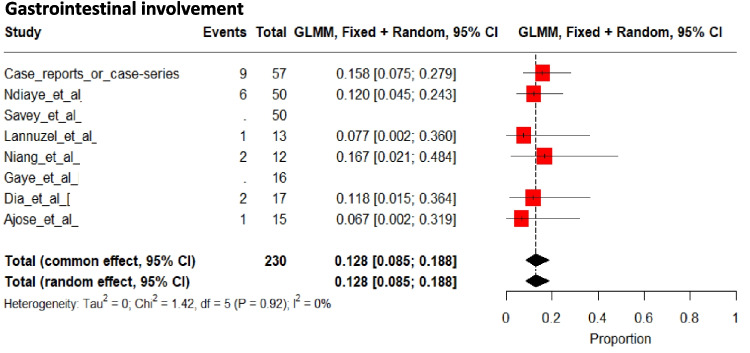
Pooled frequencies of gastro-intestinal involvement related to Behçet’s disease in adult individuals of sub-Saharan ancestry

Gastrointestinal involvement was reported in 13% [95%CI; 9 to 19] of patients (Fig. 12) [20, 29, 34, 42, 43, 48, 49, 55, 56, 61, 62, 65], and was characterized by abdominal pain in 15 patients [29, 42, 43, 48, 55, 61, 62, 65], hemorrhage in 4 patients [43, 61], and diarrhea in 2 patients [61, 65]. Details were lacking for 10 patients.

### Other Organ/System Involvement: Respiratory, Cardiac and Urogenital

Dyspnea was reported in 8 (3%) patients [32, 33, 51, 53, 63–65], pleural effusion of non-cardiac origin in 2 patients, pericardial effusion in 3, and heart failure in 1. Epididymitis was reported in five patients (2%). Renal involvement was described in three (1%) patients who presented with hematuria. No cases of renal failure were reported.

### Treatment and Outcomes

#### Overall Disease Treatments and Outcomes

Considering all studies without regard to specific organ involvement, patients were mostly treated with oral steroids (134 of 160 patients with available data, 84%), colchicine (109 patients, 68%), intravenous steroid pulses (40 patients, 9%), and azathioprine (29 patients, 18%). Other medications were less frequently prescribed (cyclophosphamide in 12 patients, dapsone in 8 patients, and methotrexate and cyclosporine in 6 patients). Anti-TNF therapy (infliximab) were prescribed to four (3%) patients [40, 43, 52, 53]. Immunosuppressive agents were used eleven times in sub-Saharan countries (azathioprine (n = 5); methotrexate (n = 4); cyclophosphamide (n = 1); cyclosporine (n = 1)). Infliximab was not prescribed in this region of the world.

Overall, 11 deaths were reported (5%) [36, 43, 55, 59, 64]. Two patients were from Senegal, 1 from Mayotte, and 6 other patients from sub-Saharan Africa (the country of origin was unknown) died in France. The 2 other death occurred in USA (n = 1, African American), and in the UK (African Caribbean from Jamaica). Regarding the causes of death, two patients died from vascular involvement (multifocal pulmonary arterial thrombosis, occlusive thrombosis of the distal aorta to renal arteries, and a refusal to benefit from thrombectomy), six without precision, three from rhombencephalitis in the context of neuro-Behçet. Finally, one patient had neuro-Behçet and died from infection.

#### Treatment for Ocular Involvement

Details about the treatment specifically initiated for ocular involvement were available in 9 reports, totaling 22 patients [35, 42–44, 52, 53, 55, 60, 66]. Two patients received steroid drops and one received intraocular steroids. All but one of the patients were treated with oral steroids, with doses ranging from 0.5 to 1 mg per kg per day. Five patients received intravenous pulses of steroids, 11 received colchicine, 9 received azathioprine, 1 received methotrexate, 6 received cyclophosphamide, and 3 received infliximab. Remission was achieved in 7 patients, but a blinding disease occurred in 9/22 patients.

Eighty-seven other patients experienced ocular involvement, but the treatment they received was not identified as specifically introduced for ocular involvement. At least 3 patients received intravenous steroids, 49 received oral steroids, 3 colchicine, 3 dapsone, 1 azathioprine, and 2 cyclophosphamide. Immunosuppressive agents were used three times in sub-Saharan Africa (South-Africa) (azathioprine (n = 1), methotrexate (n = 1), cyclosporine (n = 1)). A blinding disease occurred in 13 patients (among 48 with available information regarding disease evolution).

#### Treatment for Neurological Involvement

Details of treatment specifically initiated for neuro-Behçet were available for 31 patients [36, 38, 40, 42, 43, 46, 47, 50, 55, 56, 60, 63]. All patients received oral steroids, while 13 (42%) further received intravenous steroids. Colchicine, azathioprine, cyclosporine, and cyclophosphamide were prescribed to 26, 17, five, and seven patients, respectively. Two patients benefited from anti-TNF therapy (infliximab). In this indication, immunosuppressive agents were used two times in sub-SA countries (azathioprine (n = 1), cyclophosphamide (n = 1). Anticoagulants were administered to seven patients. Remission was achieved in 28 patients, with sequelae including hemiparesis in 4, tetraparesis in 1, dysarthria in 2, ataxia in 2, cognitive trouble in 2, pseudo-bulbar palsy in 2, drowsiness in 1, and seizures in 1 patient.

#### Treatment for Vascular Involvement

Twenty-two patients received anticoagulants. Details of immunosuppressive treatments for vascular involvement were available for ten patients. Of those patients, five received azathioprine (two of whom received it as second-line therapy), two received cyclophosphamide, two received methotrexate, and one received infliximab (as first-line therapy combined with glucocorticoids). There were no details of the treatments initiated for this involvement in sub-Saharan African countries. Five patients underwent surgery (bypass and aneurysm). One patient underwent embolization for a pulmonary embolism. Two patients underwent angioplasty with stenting for occlusion of the vertebral and lower limb arteries. In two cases, thrombolysis was attempted but was unsuccessful. One patient who refused surgery for intracardiac thrombi died from hemoptysis, hypoxemia, and hypotension [64].

## Discussion

Ethnic origin is a significant factor influencing the epidemiology and phenotype of BD. The characteristics of this systemic inflammatory disease have been poorly understood in people of sub-SA origin. Overall, this systematic review and meta-analysis found that, although BD is widely distributed, it may be less frequent in patients of sub-SA than in populations of other ancestry [1], but generally presents with severe manifestations, particularly neurological involvement. Similarly to populations of other ancestry, BD was predominantly diagnosed in males within their thirties [1]; however BD was predominantly HLA-B51-negative in the tested population of sub-SA.

The prevalence of BD was 3/100,000 inhabitants in the Caribbean Island of Guadeloupe, which is roughly similar to that in Europe [13], but significantly lower than in Japan (16/100,000 inhabitants) [6] and Turkey (80 to 600 per 100,000 inhabitants) [2–4]. The seemingly low prevalence of BD in individuals of sub-SA is illustrated by the low prevalence of this population in multiethnic studies and case series, representing 3.7 to 8.9% in New York University Hospital I, Stanford [76], or the United States Multicentric Rheumatology Informatics System for Effectiveness Registry [77], which is lower than the prevalence in the healthy African American population living in these areas [77].

More epidemiological data from countries with larger populations are required to draw concrete conclusions regarding the prevalence of BD among individuals of sub-SA. Senegalese patients constituted nearly 50% of the patients included in this systematic review. However, it is unclear why this West African country bordering the Atlantic Ocean accounts for the majority of BD cases among people living in sub-Saharan Africa included in this systematic review. For example, Nigeria is the most populous country in Africa (approximately 223 million inhabitants in 2023), but only 18 Nigerian patients were included, compared to 96 Senegalese patients (approximately 18.6 million inhabitants in 2023). A south to north and an east to west gradient of prevalence may exist; however, the absence or paucity of cases reported in other sub-Saharan countries does not allow any conclusions to be drawn. In all reports, there was no indication of the inclusion of potential immigrants from areas with a high BD prevalence (e.g., North Africa) in the study populations, and none of the patients were reported to have a mixed ethnic background. Better access to care and increased knowledge regarding this disease in Senegal are possible explanations for a higher BD prevalence, but more epidemiological studies in sub-Saharan countries and among people of sub-SA around the world are needed. The lack of epidemiological data could also be due to the fact that studies are not conducted and/or reported, and there is a need to increase epidemiological research and reporting capacities in sub-Saharan countries.

Comparing clinical characteristics between different populations is informative, but must be performed cautiously, particularly in BD, as disease prevalence and disease manifestations may vary widely within the same population and country [1–4, 73]. Nevertheless, our comparisons consistently led to the same observation, namely that CNS involvement was more common in sub-SA than in other ethnic groups (Fig. 3 and Supplementary Table 4&5) [59, 78–80]. Further, with or without studies with a neurological focus [36, 42], CNS involvement affected at least one-third of all individuals of sub-SA.

CNS involvement is a severe manifestation of BD, associated with poor prognosis and death [1, 59, 81], particularly among men and patients of sub-SA [59]. The lack of knowledge of BD and risk of diagnostic delay in individuals of sub-SA makes drawing conclusions difficult; however, the time from the first symptoms to BD diagnosis was not statistically different between individuals of sub-SA and North-African patients in a study conducted in France [59], but was shorter compared to European patients [59, 82]. Thus, the reason for the higher prevalence of BD-related CNS involvement in sub-Saharan African patients than in others remains to be elucidated. It should be noted that autoimmune diseases are consistently reported to be more severe in sub-SA patients than in others. However, these conclusions may suffer from socioeconomic bias, and data are almost completely absent in the field of autoinflammatory diseases. Given that patients from sub-Saharan Africa living in sub-Saharan Africa, Europe or the Caribbean had approximately the same level of CNS involvement, we hypothesize a greater influence of genetics rather than a possible geographical or environmental effect.

One of the most important genetic traits influencing BD is HLA-B5/B51, with studies showing that the risk of developing BD is significantly increased in HLA-B5/B51 carriers [70]. Some organ involvements have been associated with HLA-B5/B51 positivity (e.g., genital ulcers, eye and skin involvement) [71]; however, no effect on CNS involvement was observed in a meta-analysis by Maldini et al. (RR 0.95 [95% CI 0.71 to 1.27], p = 0.72) [71]. Conversely, in this analysis, CNS involvement affected only approximately 17% of the pooled patients, and of the 74 observational study populations included, none were from sub-Saharan Africa [83]. Despite being widely distributed, HLA-B51/B5 is infrequent in healthy [84] and BD-affected individuals of sub-SA [59, 85], and other genetic factors may therefore be involved [15, 86, 87].

In-depth studies of BD in individuals of sub-SA are lacking, which is one of the major limitations of this review. HLA-B5 analysis was performed in only one-third of the included patients, and no other genetic analyses or investigations were performed. Considering the rarity of HLA-B51, a genome-wide analysis may have been helpful in unraveling the genetic determinants involved in the occurrence and manifestations of BD in individuals of sub-SA [15, 87]. However, this costly analysis may not be available in low-income countries or for low-income patients, and this socioeconomic bias must be considered when interpreting the data. Facilitating access to these cutting-edge technologies for patients with BD of sub-SA may further contribute to global advances in the knowledge of BD and its pathophysiological pathways, from disease susceptibility to CNS involvement and targeted therapeutics, and will benefit all populations.

The socio-economic limitations and barriers to healthcare, which may have led to an underestimation of the prevalence of BD in the investigated population, also potentially influenced the highly limited number of modern therapies received by patients. Indeed, the anti-TNF therapy was reportedly prescribed to only four patients [40, 43, 52, 53], despite the large number of cases of severe ocular and CNS involvement for whom this may have been beneficial. It should be noted that the majority of reports are old (1994–2022) and that the use of and access to anti-TNF alpha was less common than it is today. Also, no anti-TNF treatment has been prescribed in any sub-Saharan country, except Mayotte, part of France [43], indicating the need for better access to care. Therefore, the occurrence of sequelae must be interpreted with caution, while data on outcomes driven by modern treatments in individuals of sub-SA are urgently needed.

Data on the involvement of other organs must also be treated with caution, as no study has focused on any specific organ other than the CNS; consequently, measurement or reporting bias may alter the available data. For example, data on ocular involvement are scarce, and few studies have reported a comprehensive assessment of all three ocular segments. This may explain the low prevalence of panuveitis and of total ocular involvement in sub-SA patients compared with others [84, 88]. Other limitations stem from the study design, with a predominance of old retrospective studies [20, 34, 42, 49, 59], and a small number of patients. Furthermore, given the heterogeneity between the studies included, the pooled results should be interpreted with caution, and the details per study concerning the frequencies of clinical and biological manifestations are also given in Table 1. As other limitations, meta-regression, subgroup analyses, and sensitivity analyses were not conducted in this systematic review and meta-analysis due to the limited number of included studies. In our case, the small sample size did not justify such additional analyses. Therefore, we focused on presenting a robust pooled estimates taking account heterogeneity (random effect) when appropriate. Also, publication bias was not formally assessed due to the substantial heterogeneity among the included studies in terms of study designs (case reports combined with case series and cross-sectional studies). In the presence of high clinical and methodological heterogeneity, tools for detecting publication bias may lead to incorrect interpretations. Given this variability, assessment of publication bias would have been inconclusive or misleading. Finally, some authors from organ-specialized departments (e.g., neurology or dermatology) may not have systematically assessed all of the clinical features presented by patients, and a slight measurement bias may also have been present.

Nevertheless, this comprehensive work has yielded a wealth of data on the epidemiology of BD in sub-SA patients and provides a basis for suggesting recommendations for future research, policy makers, and clinical practice. Indeed, this systematic review and meta-analysis sheds light on the urgent need for robust epidemiological studies in these patients, from predominantly sub-Saharan African ancestry but also multiethnic countries allowing further comparison with other populations.

The primary objective of collecting more robust epidemiological data is to determine the frequency and manifestations of the disease in order to prevent adverse outcomes. Another key objective is to enhance the understanding of treating physicians regarding this disease in their populations, with the aim of mitigating the adverse effects associated with BD through prompt diagnosis and appropriate treatment. CNS involvement is a major prognostic factor in BD, and this systematic review confirms its higher frequency in sub-SA patients compared to other ethnic groups [1, 59]. However, current data are insufficient to understand the origin of this difference. There is a paucity of data on HLA B51 in this population, and large-scale studies comparing HLA B51-positive and -negative patients are lacking, but these appear crucial to better understand BD in these individuals. As previously mentioned, other genetic and non-genetic factors could also explain the higher frequency of CNS manifestations in this population. An in-depth study of this population's particularities could lead to significant findings, particularly with regard to CNS involvement in Behçet's disease.

Large-scale projects could be pursued in this direction, but would require substantial funding, which could be provided by public authorities. As part of improving research funding, it is necessary to improve access to the most effective care and treatments. This systematic review revealed that very few patients were treated with immunosuppressive agents such as cyclophosphamide, azathioprine, or TNF-alpha inhibitors, despite the occurrence of severe manifestations. Behçet's disease in patients of sub-Saharan ancestry appears to be an exciting topic with many undiscovered aspects that physicians, researchers and health authorities should address. A viable starting point could be the creation of a registry of BD cases in patients of African descent based on harmonized, standardized, and potentially culturally-tailored methods.

## Conclusion

In conclusion, this systematic review with meta-analysis showed that BD is more severe among patients of sub-SA than in other ethnicities, owing to a high prevalence of CNS involvement. Therefore, individuals of sub-SA deserve particular attention at diagnosis and during follow-up. Further, we found that BD in this population is predominantly HLA B51/B5-negative. Thus, further epidemiological but also genetic studies are needed in this population to decipher the pathophysiological pathways involved, in order to potentially identify predictive factors and adapt therapeutics in at-risk patients.

## Supplementary Information

Below is the link to the electronic supplementary material.

Supplementary file1 (PDF 655 KB)

Supplementary file2 (PDF 67 KB)

Supplementary file3 (DOCX 14 KB)

Supplementary file4 (DOCX 161 KB)

Supplementary file5 (XLSX 21 KB)

## Data Availability

No datasets were generated or analysed during the current study.
