# Supplementary material for: Behçet’s Disease In Children And Adults Of Sub-Saharan Ancestry: A Systematic Review And Meta-Analysis
Source: Clin Rev Allergy Immunol. 2025 Aug 14;68(1):81. doi: 10.1007/s12016-025-09085-8 (PMC12354617; doi:10.1007/s12016-025-09085-8)
Supplement: Supplementary file 1 — Supplementary file1 (PDF 655 KB) [file 12016_2025_9085_MOESM1_ESM.pdf]

JBI CRITICAL APPRAISAL CHECKLIST FOR CASE REPORTS

Reviewer________BS AF EDF FLS__________________________________Date_________September 2023

Author____________Makgotloe__Year_2016

	Yes	No	Unclear	Not applicable
1. Were patient’s demographic characteristics clearly described?		■		☐
2. Was the patient’s history clearly described and presented as a timeline?	■		☐	☐
3. Was the current clinical condition of the patient on presentation clearly described?		☐	■	☐
4. Were diagnostic tests or assessment methods and the results clearly described?	■	☐	☐	☐
5. Was the intervention(s) or treatment procedure(s) clearly described?	■	☐	☐	☐
6. Was the post-intervention clinical condition clearly described?	■		☐	☐
7. Were adverse events (harms) or unanticipated events identified and described?			☐	■
8. Does the case report provide takeaway lessons?	■			☐

Overall appraisal: Include ■ Exclude ☐ Seek further info ☐

Comments (Including reason for exclusion)

____

____

____

____

JBI CRITICAL APPRAISAL CHECKLIST FOR CASE REPORTS

Reviewer________BS AF EDF FLS____________________________________Date__________September 2023

Author____________Meda__Year_2014

	Yes	No	Unclear	Not applicable
1. Were patient's demographic characteristics clearly described?		■		□
2. Was the patient's history clearly described and presented as a timeline?	■		□	□
3. Was the current clinical condition of the patient on presentation clearly described?	■	□		□
4. Were diagnostic tests or assessment methods and the results clearly described?	■	□	□	□
5. Was the intervention(s) or treatment procedure(s) clearly described?	■	□	□	□
6. Was the post-intervention clinical condition clearly described?	■		□	□
7. Were adverse events (harms) or unanticipated events identified and described?			□	■
8. Does the case report provide takeaway lessons?	■			□

Overall appraisal: Include ■ Exclude □ Seek further info □

Comments (Including reason for exclusion)

____

____

____

____

JBI CRITICAL APPRAISAL CHECKLIST FOR CASE REPORTS

Reviewer________BS AF EDF FLS_______________________________________Date__________September 2023

Author____________Melillo__Year_2006

	Yes	No	Unclear	Not applicable
1. Were patient's demographic characteristics clearly described?		■		☐
2. Was the patient's history clearly described and presented as a timeline?	■		☐	☐
3. Was the current clinical condition of the patient on presentation clearly described?	■	☐		☐
4. Were diagnostic tests or assessment methods and the results clearly described?	■	☐	☐	☐
5. Was the intervention(s) or treatment procedure(s) clearly described?	■	☐	☐	☐
6. Was the post-intervention clinical condition clearly described?	■		☐	☐
7. Were adverse events (harms) or unanticipated events identified and described?			☐	■
8. Does the case report provide takeaway lessons?	■			☐

Overall appraisal: Include ■ Exclude ☐ Seek further info ☐

Comments (Including reason for exclusion)

____

____

____

____

JBI CRITICAL APPRAISAL CHECKLIST FOR CASE REPORTS

Reviewer________BS AF EDF FLS___________________________________Date__________September 2023

Author____________Merkler__Year_2015

	Yes	No	Unclear	Not applicable
1. Were patient's demographic characteristics clearly described?		■		☐
2. Was the patient's history clearly described and presented as a timeline?	■		☐	☐
3. Was the current clinical condition of the patient on presentation clearly described?	■	☐		☐
4. Were diagnostic tests or assessment methods and the results clearly described?	■	☐	☐	☐
5. Was the intervention(s) or treatment procedure(s) clearly described?		■	☐	☐
6. Was the post-intervention clinical condition clearly described?		■	☐	☐
7. Were adverse events (harms) or unanticipated events identified and described?			☐	■
8. Does the case report provide takeaway lessons?			■	☐

Overall appraisal: Include ■ Exclude ☐ Seek further info ☐

Comments (Including reason for exclusion)

____

____

____

____

JBI CRITICAL APPRAISAL CHECKLIST FOR CASE REPORTS

Reviewer________BS AF EDF FLS___________________________________Date__________September 2023

Author____________Mitra__Year_2015

	Yes	No	Unclear	Not applicable
1. Were patient's demographic characteristics clearly described?		■		☐
2. Was the patient's history clearly described and presented as a timeline?		■	☐	☐
3. Was the current clinical condition of the patient on presentation clearly described?	■	☐		☐
4. Were diagnostic tests or assessment methods and the results clearly described?	■	☐	☐	☐
5. Was the intervention(s) or treatment procedure(s) clearly described?	■		☐	☐
6. Was the post-intervention clinical condition clearly described?	■		☐	☐
7. Were adverse events (harms) or unanticipated events identified and described?			☐	■
8. Does the case report provide takeaway lessons?	■			☐

Overall appraisal: Include ■ Exclude ☐ Seek further info ☐

Comments (Including reason for exclusion)

JBI CRITICAL APPRAISAL CHECKLIST FOR CASE REPORTS

Reviewer________BS AF EDF FLS___________________________________Date__________September 2023

Author____________Nkam__Year_2006

	Yes	No	Unclear	Not applicable
1. Were patient's demographic characteristics clearly described?	■			☐
2. Was the patient's history clearly described and presented as a timeline?	■		☐	☐
3. Was the current clinical condition of the patient on presentation clearly described?	■	☐		☐
4. Were diagnostic tests or assessment methods and the results clearly described?	■	☐	☐	☐
5. Was the intervention(s) or treatment procedure(s) clearly described?	■		☐	☐
6. Was the post-intervention clinical condition clearly described?	■		☐	☐
7. Were adverse events (harms) or unanticipated events identified and described?	■		☐	
8. Does the case report provide takeaway lessons?	■			☐

Overall appraisal: Include ■ Exclude ☐ Seek further info ☐

Comments (Including reason for exclusion)

____

____

____

____

JBI CRITICAL APPRAISAL CHECKLIST FOR CASE REPORTS

Reviewer________BS AF EDF FLS___________________________________Date__________September 2023

Author____________Nokes__Year_2018

	Yes	No	Unclear	Not applicable
1. Were patient's demographic characteristics clearly described?	■			☐
2. Was the patient's history clearly described and presented as a timeline?	■		☐	☐
3. Was the current clinical condition of the patient on presentation clearly described?	■	☐		☐
4. Were diagnostic tests or assessment methods and the results clearly described?	■	☐	☐	☐
5. Was the intervention(s) or treatment procedure(s) clearly described?	■		☐	☐
6. Was the post-intervention clinical condition clearly described?	■		☐	☐
7. Were adverse events (harms) or unanticipated events identified and described?	■		☐	
8. Does the case report provide takeaway lessons?	■			☐

Overall appraisal: Include ■ Exclude ☐ Seek further info ☐

Comments (Including reason for exclusion)

JBI CRITICAL APPRAISAL CHECKLIST FOR CASE REPORTS

Reviewer________BS AF EDF FLS____________________________________Date__________September 2023

Author____________O'Leary__Year_2011

	Yes	No	Unclear	Not applicable
1. Were patient's demographic characteristics clearly described?		■		☐
2. Was the patient's history clearly described and presented as a timeline?	■		☐	☐
3. Was the current clinical condition of the patient on presentation clearly described?	■	☐		☐
4. Were diagnostic tests or assessment methods and the results clearly described?	■	☐	☐	☐
5. Was the intervention(s) or treatment procedure(s) clearly described?	■		☐	☐
6. Was the post-intervention clinical condition clearly described?		■	☐	☐
7. Were adverse events (harms) or unanticipated events identified and described?	■		☐	
8. Does the case report provide takeaway lessons?	■			☐

Overall appraisal: Include ■ Exclude ☐ Seek further info ☐

Comments (Including reason for exclusion)

____

____

____

____

JBI CRITICAL APPRAISAL CHECKLIST FOR CASE REPORTS

Reviewer_______BS AF EDF FLS_________________________________Date_________September 2023

Author___________Pandrea_Year_2007

	Yes	No	Unclear	Not applicable
1. Were patient's demographic characteristics clearly described?	■			☐
2. Was the patient's history clearly described and presented as a timeline?	■		☐	☐
3. Was the current clinical condition of the patient on presentation clearly described?	■	☐		☐
4. Were diagnostic tests or assessment methods and the results clearly described?	■	☐	☐	☐
5. Was the intervention(s) or treatment procedure(s) clearly described?	■		☐	☐
6. Was the post-intervention clinical condition clearly described?	■		☐	☐
7. Were adverse events (harms) or unanticipated events identified and described?	■		☐	
8. Does the case report provide takeaway lessons?	■			☐

Overall appraisal: Include ■ Exclude ☐ Seek further info ☐

Comments (Including reason for exclusion)

___

___

___

___

JBI CRITICAL APPRAISAL CHECKLIST FOR CASE REPORTS

Reviewer________BS AF EDF FLS___________________________________Date__________September 2023

Author____________Patel__Year_2012

	Yes	No	Unclear	Not applicable
1. Were patient's demographic characteristics clearly described?		■		☐
2. Was the patient's history clearly described and presented as a timeline?	■		☐	☐
3. Was the current clinical condition of the patient on presentation clearly described?	■	☐		☐
4. Were diagnostic tests or assessment methods and the results clearly described?	■	☐	☐	☐
5. Was the intervention(s) or treatment procedure(s) clearly described?		■	☐	☐
6. Was the post-intervention clinical condition clearly described?		■	☐	☐
7. Were adverse events (harms) or unanticipated events identified and described?			☐	■
8. Does the case report provide takeaway lessons?	■			☐

Overall appraisal: Include ■ Exclude ☐ Seek further info ☐

Comments (Including reason for exclusion)

___

___

___

___

JBI Critical Appraisal Checklist for Case Series

Reviewer____________BS____AF____FLS____EDF_____________________________________Date______September 2023___________________________

Author________Poon______________________________________Year_____2003

	Yes	No	Unclear	Not applicable
• Were there clear criteria for inclusion in the case series?		■	☐	☐
• Was the condition measured in a standard, reliable way for all participants included in the case series?	■	☐	☐	☐
• Were valid methods used for identification of the condition for all participants included in the case series?		☐	■	☐
• Did the case series have consecutive inclusion of participants?			■	☐
• Did the case series have complete inclusion of participants?	☐	☐	■	☐
• Was there clear reporting of the demographics of the participants in the study?	■	☐	☐	☐
• Was there clear reporting of clinical information of the participants?	■	☐	☐	☐
• Were the outcomes or follow up results of cases clearly reported?	■	☐	☐	☐
• Was there clear reporting of the presenting site(s)/clinic(s) demographic information?	■	☐	☐	☐
• Was statistical analysis appropriate?	☐	☐	☐	■

Overall appraisal: Include ■ Exclude ☐ Seek further info ☐

Comments (Including reason for exclusion)

JBI CRITICAL APPRAISAL CHECKLIST FOR CASE REPORTS

Reviewer________BS AF EDF FLS___________________________________Date__________September 2023

Author____________Pretorius__Year_2022

	Yes	No	Unclear	Not applicable
1. Were patient's demographic characteristics clearly described?		■		☐
2. Was the patient's history clearly described and presented as a timeline?	■		☐	☐
3. Was the current clinical condition of the patient on presentation clearly described?	■	☐		☐
4. Were diagnostic tests or assessment methods and the results clearly described?	■	☐	☐	☐
5. Was the intervention(s) or treatment procedure(s) clearly described?	■		☐	☐
6. Was the post-intervention clinical condition clearly described?			■	☐
7. Were adverse events (harms) or unanticipated events identified and described?	■		☐	
8. Does the case report provide takeaway lessons?	■			☐

Overall appraisal: Include ■ Exclude ☐ Seek further info ☐

Comments (Including reason for exclusion)

____

____

____

____

JBI CRITICAL APPRAISAL CHECKLIST FOR CASE REPORTS

Reviewer________BS AF EDF FLS___________________________________Date__________September 2023

Author____________Rao__Year_2015

	Yes	No	Unclear	Not applicable
1. Were patient's demographic characteristics clearly described?		■		☐
2. Was the patient's history clearly described and presented as a timeline?	■		☐	☐
3. Was the current clinical condition of the patient on presentation clearly described?	■	☐		☐
4. Were diagnostic tests or assessment methods and the results clearly described?	■	☐	☐	☐
5. Was the intervention(s) or treatment procedure(s) clearly described?	■		☐	☐
6. Was the post-intervention clinical condition clearly described?	■			☐
7. Were adverse events (harms) or unanticipated events identified and described?	■		☐	
8. Does the case report provide takeaway lessons?	■			☐

Overall appraisal: Include ■ Exclude ☐ Seek further info ☐

Comments (Including reason for exclusion)

____

____

____

____

JBI CRITICAL APPRAISAL CHECKLIST FOR CASE REPORTS

Reviewer________BS AF EDF FLS____________________________________Date__________September 2023

Author_____________Sarr__Year_2015

	Yes	No	Unclear	Not applicable
1. Were patient's demographic characteristics clearly described?		■		☐
2. Was the patient's history clearly described and presented as a timeline?		■	☐	☐
3. Was the current clinical condition of the patient on presentation clearly described?	■	☐		☐
4. Were diagnostic tests or assessment methods and the results clearly described?	■	☐	☐	☐
5. Was the intervention(s) or treatment procedure(s) clearly described?	■		☐	☐
6. Was the post-intervention clinical condition clearly described?		■		☐
7. Were adverse events (harms) or unanticipated events identified and described?			☐	■
8. Does the case report provide takeaway lessons?	■			☐

Overall appraisal: Include ■ Exclude ☐ Seek further info ☐

Comments (Including reason for exclusion)

JBI CRITICAL APPRAISAL CHECKLIST FOR CASE REPORTS

Reviewer________BS AF EDF FLS____________________________________Date__________September 2023

Author_____________Savini__Year_2008

	Yes	No	Unclear	Not applicable
1. Were patient's demographic characteristics clearly described?	■			☐
2. Was the patient's history clearly described and presented as a timeline?	■		☐	☐
3. Was the current clinical condition of the patient on presentation clearly described?	■	☐		☐
4. Were diagnostic tests or assessment methods and the results clearly described?	■	☐	☐	☐
5. Was the intervention(s) or treatment procedure(s) clearly described?	■		☐	☐
6. Was the post-intervention clinical condition clearly described?		■		☐
7. Were adverse events (harms) or unanticipated events identified and described?			☐	■
8. Does the case report provide takeaway lessons?	■			☐

Overall appraisal: Include ■ Exclude ☐ Seek further info ☐

Comments (Including reason for exclusion)

JBI CRITICAL APPRAISAL CHECKLIST FOR CASE REPORTS

Reviewer_______BS AF EDF FLS_________________________________Date_________September 2023

Author____________Smith__Year_2002

	Yes	No	Unclear	Not applicable
1. Were patient's demographic characteristics clearly described?	■			☐
2. Was the patient's history clearly described and presented as a timeline?	■		☐	☐
3. Was the current clinical condition of the patient on presentation clearly described?	■	☐		☐
4. Were diagnostic tests or assessment methods and the results clearly described?	■	☐	☐	☐
5. Was the intervention(s) or treatment procedure(s) clearly described?	■		☐	☐
6. Was the post-intervention clinical condition clearly described?		■		☐
7. Were adverse events (harms) or unanticipated events identified and described?			☐	■
8. Does the case report provide takeaway lessons?	■			☐

Overall appraisal: Include ■ Exclude ☐ Seek further info ☐

Comments (Including reason for exclusion)

____

____

____

____

JBI CRITICAL APPRAISAL CHECKLIST FOR CASE REPORTS

Reviewer________BS AF EDF FLS___________________________________Date__________September 2023

Author____________Taylor__Year_1997

		Yes	No	Unclear	Not applicable
1.	Were patient's demographic characteristics clearly described?	■			☐
2.	Was the patient's history clearly described and presented as a timeline?	■		☐	☐
3.	Was the current clinical condition of the patient on presentation clearly described?	■	☐		☐
4.	Were diagnostic tests or assessment methods and the results clearly described?	■	☐	☐	☐
5.	Was the intervention(s) or treatment procedure(s) clearly described?	■		☐	☐
6.	Was the post-intervention clinical condition clearly described?	■			☐
7.	Were adverse events (harms) or unanticipated events identified and described?	■		☐	
8.	Does the case report provide takeaway lessons?	■			☐

Overall appraisal: Include ■ Exclude ☐ Seek further info ☐

Comments (Including reason for exclusion)

____

____

____

____

JBI CRITICAL APPRAISAL CHECKLIST FOR CASE REPORTS

Reviewer________BS AF EDF FLS___________________________________Date__________September 2023

Author____________Watkins__Year_2018

	Yes	No	Unclear	Not applicable
1. Were patient's demographic characteristics clearly described?	■			☐
2. Was the patient's history clearly described and presented as a timeline?	■		☐	☐
3. Was the current clinical condition of the patient on presentation clearly described?	■	☐		☐
4. Were diagnostic tests or assessment methods and the results clearly described?	■	☐	☐	☐
5. Was the intervention(s) or treatment procedure(s) clearly described?	■		☐	☐
6. Was the post-intervention clinical condition clearly described?	■			☐
7. Were adverse events (harms) or unanticipated events identified and described?	■		☐	
8. Does the case report provide takeaway lessons?	■			☐

Overall appraisal: Include ■ Exclude ☐ Seek further info ☐

Comments (Including reason for exclusion)

____

____

____

____

JBI CRITICAL APPRAISAL CHECKLIST FOR CASE REPORTS

Reviewer________BS AF EDF FLS___________________________________Date__________September 2023

Author____________Winer-Muram__Year_1994

	Yes	No	Unclear	Not applicable
1. Were patient's demographic characteristics clearly described?		■		☐
2. Was the patient's history clearly described and presented as a timeline?		■	☐	☐
3. Was the current clinical condition of the patient on presentation clearly described?	■	☐		☐
4. Were diagnostic tests or assessment methods and the results clearly described?	■	☐	☐	☐
5. Was the intervention(s) or treatment procedure(s) clearly described?	■		☐	☐
6. Was the post-intervention clinical condition clearly described?	■			☐
7. Were adverse events (harms) or unanticipated events identified and described?	■		☐	
8. Does the case report provide takeaway lessons?	■			☐

Overall appraisal: Include ■ Exclude ☐ Seek further info ☐

Comments (Including reason for exclusion)

JBI CRITICAL APPRAISAL CHECKLIST FOR CASE REPORTS

Reviewer________BS AF EDF FLS____________________________________Date__________September 2023_________________________

Author____________Ali Munive________________________________Year_2001_________

	Yes	No	Unclear	Not applicable
1. Were patient's demographic characteristics clearly described?	■	☐	☐	☐
2. Was the patient's history clearly described and presented as a timeline?	■	☐	☐	☐
3. Was the current clinical condition of the patient on presentation clearly described?	■	☐	☐	☐
4. Were diagnostic tests or assessment methods and the results clearly described?	■	☐	☐	☐
5. Was the intervention(s) or treatment procedure(s) clearly described?	■	☐	☐	☐
6. Was the post-intervention clinical condition clearly described?	■	☐	☐	☐
7. Were adverse events (harms) or unanticipated events identified and described?	☐	■	☐	☐
8. Does the case report provide takeaway lessons?	☐	☐	■	☐

Overall appraisal: Include ■ Exclude ☐ Seek further info ☐

Comments (Including reason for exclusion)

JBI CRITICAL APPRAISAL CHECKLIST FOR CASE REPORTS

Reviewer________BS AF EDF FLS____________________________________Date__________September 2023__________________________

Author____________Angotti________________________________Year_2003_________

	Yes	No	Unclear	Not applicable
1. Were patient's demographic characteristics clearly described?		☐	■	☐
2. Was the patient's history clearly described and presented as a timeline?	■	☐	☐	☐
3. Was the current clinical condition of the patient on presentation clearly described?	■	☐	☐	☐
4. Were diagnostic tests or assessment methods and the results clearly described?	■	☐	☐	☐
5. Was the intervention(s) or treatment procedure(s) clearly described?	■	☐	☐	☐
6. Was the post-intervention clinical condition clearly described?		■	☐	☐
7. Were adverse events (harms) or unanticipated events identified and described?	■		☐	☐
8. Does the case report provide takeaway lessons?	☐	■		☐

Overall appraisal: Include ■ Exclude ☐ Seek further info ☐

Comments (Including reason for exclusion)

____

JBI CRITICAL APPRAISAL CHECKLIST FOR CASE REPORTS

Reviewer________BS AF EDF FLS___________________________________Date__________November 2024_________________________

Author____________Awoyesuku__Year_2010_________

	Yes	No	Unclear	Not applicable
1. Were patient's demographic characteristics clearly described?	■	☐		☐
2. Was the patient's history clearly described and presented as a timeline?	■	☐	☐	☐
3. Was the current clinical condition of the patient on presentation clearly described?	■	☐	☐	☐
4. Were diagnostic tests or assessment methods and the results clearly described?	■	☐	☐	☐
5. Was the intervention(s) or treatment procedure(s) clearly described?	■	☐	☐	☐
6. Was the post-intervention clinical condition clearly described?		■	☐	☐
7. Were adverse events (harms) or unanticipated events identified and described?	■		☐	☐
8. Does the case report provide takeaway lessons?	■			☐

Overall appraisal: Include ■ Exclude ☐ Seek further info ☐

Comments (Including reason for exclusion)

____

____

____

____

JBI CRITICAL APPRAISAL CHECKLIST FOR CASE REPORTS

Reviewer________BS AF EDF FLS___________________________________Date__________September 2023

Author____________Ba Djibril__Year_2016

	Yes	No	Unclear	Not applicable
1. Were patient's demographic characteristics clearly described?	■	☐		☐
2. Was the patient's history clearly described and presented as a timeline?	■	☐	☐	☐
3. Was the current clinical condition of the patient on presentation clearly described?	■	☐	☐	☐
4. Were diagnostic tests or assessment methods and the results clearly described?	■	☐	☐	☐
5. Was the intervention(s) or treatment procedure(s) clearly described?	■	☐	☐	☐
6. Was the post-intervention clinical condition clearly described?		■	☐	☐
7. Were adverse events (harms) or unanticipated events identified and described?	■		☐	☐
8. Does the case report provide takeaway lessons?	■			☐

Overall appraisal: Include ■ Exclude ☐ Seek further info ☐

Comments (Including reason for exclusion)

____

____

____

____

JBI CRITICAL APPRAISAL CHECKLIST FOR CASE REPORTS

Reviewer________BS AF EDF FLS____________________________________Date__________September 2023

Author____________Carvalho__Year_2011

	Yes	No	Unclear	Not applicable
1. Were patient's demographic characteristics clearly described?		■		☐
2. Was the patient's history clearly described and presented as a timeline?	■	☐	☐	☐
3. Was the current clinical condition of the patient on presentation clearly described?	■	☐	☐	☐
4. Were diagnostic tests or assessment methods and the results clearly described?	■	☐	☐	☐
5. Was the intervention(s) or treatment procedure(s) clearly described?	■	☐	☐	☐
6. Was the post-intervention clinical condition clearly described?		■	☐	☐
7. Were adverse events (harms) or unanticipated events identified and described?			☐	■
8. Does the case report provide takeaway lessons?	■			☐

Overall appraisal: Include ■ Exclude ☐ Seek further info ☐

Comments (Including reason for exclusion)

____

____

____

____

JBI CRITICAL APPRAISAL CHECKLIST FOR CASE REPORTS

Reviewer_______BS AF EDF FLS_________________________________Date_________September 2023

Author___________Ecker__Year_2000

	Yes	No	Unclear	Not applicable
1. Were patient's demographic characteristics clearly described?		■		☐
2. Was the patient's history clearly described and presented as a timeline?	■	☐	☐	☐
3. Was the current clinical condition of the patient on presentation clearly described?	■	☐	☐	☐
4. Were diagnostic tests or assessment methods and the results clearly described?	■	☐	☐	☐
5. Was the intervention(s) or treatment procedure(s) clearly described?	■	☐	☐	☐
6. Was the post-intervention clinical condition clearly described?		■	☐	☐
7. Were adverse events (harms) or unanticipated events identified and described?			☐	■
8. Does the case report provide takeaway lessons?		■		☐

Overall appraisal: Include ■ Exclude ☐ Seek further info ☐

Comments (Including reason for exclusion)

____

____

____

____

JBI CRITICAL APPRAISAL CHECKLIST FOR CASE REPORTS

Reviewer________BS AF EDF FLS____________________________________Date__________September 2023

Author____________Getachew__Year_2020

	Yes	No	Unclear	Not applicable
1. Were patient's demographic characteristics clearly described?	■			☐
2. Was the patient's history clearly described and presented as a timeline?	■	☐	☐	☐
3. Was the current clinical condition of the patient on presentation clearly described?	■	☐	☐	☐
4. Were diagnostic tests or assessment methods and the results clearly described?	■	☐	☐	☐
5. Was the intervention(s) or treatment procedure(s) clearly described?	■	☐	☐	☐
6. Was the post-intervention clinical condition clearly described?		■	☐	☐
7. Were adverse events (harms) or unanticipated events identified and described?	■		☐	
8. Does the case report provide takeaway lessons?	■			☐

Overall appraisal: Include ■ Exclude ☐ Seek further info ☐

Comments (Including reason for exclusion)

____

____

____

____

JBI CRITICAL APPRAISAL CHECKLIST FOR CASE REPORTS

Reviewer________BS AF EDF FLS____________________________________Date__________November 2024

Author____________Ikuabe__Year_2014

	Yes	No	Unclear	Not applicable
1. Were patient's demographic characteristics clearly described?	■			☐
2. Was the patient's history clearly described and presented as a timeline?	■	☐	☐	☐
3. Was the current clinical condition of the patient on presentation clearly described?	■	☐	☐	☐
4. Were diagnostic tests or assessment methods and the results clearly described?	■	☐	☐	☐
5. Was the intervention(s) or treatment procedure(s) clearly described?	■	☐	☐	☐
6. Was the post-intervention clinical condition clearly described?		■	☐	☐
7. Were adverse events (harms) or unanticipated events identified and described?			☐	■
8. Does the case report provide takeaway lessons?	■			☐

Overall appraisal: Include ■ Exclude ☐ Seek further info ☐

Comments (Including reason for exclusion)

____

____

____

____

JBI CRITICAL APPRAISAL CHECKLIST FOR CASE REPORTS

Reviewer________BS AF EDF FLS___________________________________Date__________September 2023

Author____________Jacyk__Year_1994

	Yes	No	Unclear	Not applicable
1. Were patient's demographic characteristics clearly described?			■	☐
2. Was the patient's history clearly described and presented as a timeline?			■	☐
3. Was the current clinical condition of the patient on presentation clearly described?	■	☐		☐
4. Were diagnostic tests or assessment methods and the results clearly described?	■	☐	☐	☐
5. Was the intervention(s) or treatment procedure(s) clearly described?	■		☐	☐
6. Was the post-intervention clinical condition clearly described?			■	☐
7. Were adverse events (harms) or unanticipated events identified and described?			■	
8. Does the case report provide takeaway lessons?	■			☐

Overall appraisal: Include ■ Exclude ☐ Seek further info ☐

Comments (Including reason for exclusion)

___

___

___

___

JBI CRITICAL APPRAISAL CHECKLIST FOR CASE REPORTS

Reviewer________BS AF EDF FLS___________________________________Date__________November 2024

Author____________Kasirajan__Year_2001

	Yes	No	Unclear	Not applicable
1. Were patient's demographic characteristics clearly described?		■		☐
2. Was the patient's history clearly described and presented as a timeline?	■	☐	☐	☐
3. Was the current clinical condition of the patient on presentation clearly described?	■	☐	☐	☐
4. Were diagnostic tests or assessment methods and the results clearly described?	■	☐	☐	☐
5. Was the intervention(s) or treatment procedure(s) clearly described?	■	☐	☐	☐
6. Was the post-intervention clinical condition clearly described?	■		☐	☐
7. Were adverse events (harms) or unanticipated events identified and described?	■		☐	
8. Does the case report provide takeaway lessons?	■			☐

Overall appraisal: Include ■ Exclude ☐ Seek further info ☐

Comments (Including reason for exclusion)

___

___

___

___

JBI CRITICAL APPRAISAL CHECKLIST FOR CASE REPORTS

Reviewer_______BS AF EDF FLS_________________________________Date_________September 2023

Author___________Khoo__Year_2021

	Yes	No	Unclear	Not applicable
1. Were patient's demographic characteristics clearly described?	■			□
2. Was the patient's history clearly described and presented as a timeline?	■	□	□	□
3. Was the current clinical condition of the patient on presentation clearly described?	■	□	□	□
4. Were diagnostic tests or assessment methods and the results clearly described?	■	□	□	□
5. Was the intervention(s) or treatment procedure(s) clearly described?	■	□	□	□
6. Was the post-intervention clinical condition clearly described?	■		□	□
7. Were adverse events (harms) or unanticipated events identified and described?			□	■
8. Does the case report provide takeaway lessons?	■			□

Overall appraisal: Include ■ Exclude □ Seek further info □

Comments (Including reason for exclusion)

____

____

____

____

JBI CRITICAL APPRAISAL CHECKLIST FOR CASE REPORTS

Reviewer________BS AF EDF FLS__________________________________Date__________September 2023

Author____________Kurada__Year_2023

	Yes	No	Unclear	Not applicable
1. Were patient's demographic characteristics clearly described?	■			☐
2. Was the patient's history clearly described and presented as a timeline?		■	☐	☐
3. Was the current clinical condition of the patient on presentation clearly described?		☐	■	☐
4. Were diagnostic tests or assessment methods and the results clearly described?	■	☐	☐	☐
5. Was the intervention(s) or treatment procedure(s) clearly described?	■	☐	☐	☐
6. Was the post-intervention clinical condition clearly described?		■	☐	☐
7. Were adverse events (harms) or unanticipated events identified and described?		■	☐	
8. Does the case report provide takeaway lessons?	■			☐

Overall appraisal: Include ■ Exclude ☐ Seek further info ☐

Comments (Including reason for exclusion)

____

____

____

____

JBI Critical Appraisal Checklist for Case Series

Reviewer____________BS AF FLS EDF____Date______September 2023_____________________________

Author_________Liozon______________________________________Year_____2011

	Yes	No	Unclear	Not applicable
• Were there clear criteria for inclusion in the case series?	■	☐	☐	☐
• Was the condition measured in a standard, reliable way for all participants included in the case series?	■	☐	☐	☐
• Were valid methods used for identification of the condition for all participants included in the case series?	■	☐	☐	☐
• Did the case series have consecutive inclusion of participants?	■			☐
• Did the case series have complete inclusion of participants?	☐	☐	■	☐
• Was there clear reporting of the demographics of the participants in the study?	■	☐	☐	☐
• Was there clear reporting of clinical information of the participants?	■	☐	☐	☐
• Were the outcomes or follow up results of cases clearly reported?	■	☐	☐	☐
• Was there clear reporting of the presenting site(s)/clinic(s) demographic information?	■	☐	☐	☐
• Was statistical analysis appropriate?	☐	☐	☐	■

Overall appraisal: Include ■ Exclude ☐ Seek further info ☐

Comments (Including reason for exclusion)
