# Supplementary material for: Behçet’s Disease In Children And Adults Of Sub-Saharan Ancestry: A Systematic Review And Meta-Analysis
Source: Clin Rev Allergy Immunol. 2025 Aug 14;68(1):81. doi: 10.1007/s12016-025-09085-8 (PMC12354617; doi:10.1007/s12016-025-09085-8)
Supplement: Supplementary file 3 — Supplementary file3 (DOCX 14 KB) [file 12016_2025_9085_MOESM3_ESM.docx]

**Supplemental material 2: Behçet’s disease in adults of sub-Saharan ancestry (systematic review and meta-analysis): Use of immunosuppressive treatment**

Azathioprine: 1 patient following cyclophosphamide for CNS disease (Lannuzel)

3 patients received and “responded” to azathioprine without knowledge about the reason of introduction (Ajose)

5 patients for ocular involvement (first line (n=1+1 (+CNS disease) + 1); unknown line (n=1+); second line (n=1) (Poon)

8 patients unknown reason, unknown involvement, unknown sequence (Liozon)

1 patient for neuropsychiatric reason, first line with colchicine and steroids (Nkam)

1 patient for vascular disease, first line, alone (Taylor)

1 patient for vascular disease, first line, with steroids without colchicine (Kurada)

1 patient for CNS disease, IV GC then oral GC + azathioprine as bi therapy first line (Watkins)

1 patient for ocular disease, AZA + IFX + GC as tritherapy first line (Pandrea)

2 patients for vascular disease, as second line therapy following oral GC (Nokes)

1 patient for **oral ulcers** as second line therapy (Angotti)

1 patient (100 mg/d) for vascular disease, first line therapy along with GC 1 mg/kg (Ali munive)

1 patient for ocular involvement, tritherapy with GC and cyclosporine (Makgotloe)

1 patient for vascular involvement without knowledge about sequence (Carvalho)

1 patient for CNS involvement, second line (Pretorius)

Cyclophosphamide:

3 patients as a combination therapy with colchicine and glucocorticoids for CNS disease (Lannuzel)

1 patient vascular disease (Rao)

1 patient vascular disease (Muram)

1 patient for CNS disease (Melillo)

1 patient for CNS disease, along with GC (Khoo)

1 patient for CNS disease, first line (Savini)

1 patient for CNS disease, first line (Nkam)

1 patient for CNS disease, without knowledge about sequence (Liozon)

1 patient for CNS disease, first line, along with high dose intravenous steroids (Merkler)

1 patient for CNS, first line (Pretorius)

Methotrexate:

1 patient for **oral ulcers** as third line therapy, and first line therapy vascular disease (Angotti)

3 patients, without knowledge of indication and sequence (Ajose)

1 patient for vascular involvement without knowledge about sequence (Carvalho)

1 patient for ocular involvement (Makgot)

IFX:

1 patient after cyclophosphamide for CNS disease, third line (Khoo)

1 patient for CNS disease, second line, (Liozon)

1 patient for vascular disease, first line along with steroids (O’Leary)

1 patient for ocular disease, first line along with steroids and azathioprine, for 6 months (Pandrea)

Ciclosporine:

1 patient for ocular disease, unknown line (Poon)

1 patient for ocular disease, second line, combination with GC (Poon)

1 patient for ocular disease, second line with GC + AZA (Makgo)

**Regions immunosuppressant:**

Azathioprine:

8 + 1 + 1 = France (dont Mayotte) (Liozon, Lannuzel, Nkam)

5 + 1 + 1 = UK (Poon, Taylor)

3 = Nigeria (Ajose)

1 + 1 + 1 + 1 + 1 = USA (Kurada, Watkins, Pandrea, Nokes, Angotti)

1 = Colombia (Ali Munive)

1 + 1 = South Africa (Pretorius, Makgotloe)

1 = Brazil (Carvalho)

Methotrexate:

3 = Nigeria (Ajose)

1 = Brazil (Carvalho)

1 = UK (Angotti)

1 = South Africa (Makgotloe)

CYC:

1 + 1 = UK (Melillo, Khoo)

3 + 1 + 1 + 1 = France (Lannuzel, Savini, Nkam, Liozon)

1 + 1 + 1 = USA (Merkler, Rao, Winer-Muram)

1 = South Africa (Pretorius)

Ciclosporine:

1 = South Africa (Makgotloe)

2 = UK (Poon)

Infliximab:

1 + 1 = USA (O’Leary, Pandrea)

1 = France (Liozon)

1 = UK (Khoo)
