# Supplementary material for: Behçet’s Disease In Children And Adults Of Sub-Saharan Ancestry: A Systematic Review And Meta-Analysis
Source: Clin Rev Allergy Immunol. 2025 Aug 14;68(1):81. doi: 10.1007/s12016-025-09085-8 (PMC12354617; doi:10.1007/s12016-025-09085-8)

**Epidemiology of Behçet’s disease and its manifestations in individuals of sub-Saharan ancestry: a systematic review and meta-analysis**

# **TABLE S1**: Search strategy for PubMed/Medline

|  | Search terms |
| --- | --- |
| First term | "Behcet syndrome"[MeSH Terms] OR Behcet's disease [Text Word for this term and the following] Behcet's Syndrome OR Triple-Symptom Complex OR Behcet Disease OR Behçet Disease OR Behçet Diseases OR Adamantiades-Behcet Disease OR Adamantiades Behcet Disease OR Adamantiades-Behcet Diseases OR Behcet Triple Symptom Complex OR Old Silk Route Disease OR Behcet's Disease OR Triple Symptom Complex OR Symptom Complex OR Triple OR Triple Symptom Complices |
| Second term | Black people [MeSh] OR Sub-Saharan African People [MeSh] Or Africa South of the Sahara [Mesh] OR Africa OR Black |
| Search strategy | First terms AND second terms |
|  | Database inception to September 1st, 2023. A second analysis has been performed with a search period from September 1^st^ 2023, to November 29^th^ 2024 |

# **TABLE S2**: Search strategy for African Journals Online

|  | Search terms |
| --- | --- |
| First term | "Behcet’s disease” OR Behçet’s syndrome” |
| Search strategy | First term |
|  | Database inception to November 29^th^ 2024 |

**Table S3: Systematic reviews and meta-analysis of Behçet’s disease in patients of sub-Saharan ancestry: characteristics of included primary studies. Study quality by NIHLBI study quality assessment tool**

|  | **Ndiaye et al** (1) | **Savey et al** (2) | **Lannuzel et al** (3) | **Niang et al** (4) | **Gaye et al** (5) | **Dia et al** (6) | **Ajose et al** (7) |
| --- | --- | --- | --- | --- | --- | --- | --- |
| Study period | 2000 to 2013 | 1974 to 2010 | 1989 to 1999 | 1997 to 2006 | 2012 to 2018 | 1970 to 1995 | 2007 to 2011 |
| Design | **Retrospective** | **Retrospective** | **Retrospective** | **Retrospective** | **Retrospective** | **Retrospective** | Prospective |
| Comparative study ? | No | Yes | No | No | No | No | No |
| Multicentric ? | No | No | No | No | No | Yes | No |
| Country | Senegal | France | Guadeloupe | Senegal | Senegal | Senegal | Nigeria |
| Hospital based ? | Yes | Yes | Yes | Yes | Yes | Yes | Yes |
| Department(s) | Dermatology | Internal medicine | Neurology | Dermatology | Neurology | Dermatology, Internal medicine, ophthalmology, neurology | Dermatology& rheumatology |
| Number of patients | 50 | 50 (6.5%) sub-Saharan African patients among 769 | 13 | 12 | 16 | 17 | 15 |
| Age at diagnosis | 32 | 32 | N/A | 24, mean value | 40, mean value | 28, mean value | 27 y.o. mean value |
| Time to diagnosis |  | 23 [0; 91], median value |  | 17 [1; 72],  mean value |  |  | 72 (N/A),  mean value |
| Study quality | Fair | Good | Fair | Fair | Fair | Poor | Good |

**Table S4:** Summary of the Characteristics of studies on patients with Behçet’s disease according to their region of origin

|  | **America** | **Caribbean** | **Sub-Saharan Africa** |
| --- | --- | --- | --- |
| Publication period | 1994-2023 | 2001-2015 | 1994-2022 |
| Number (n, %) | 13 | 22 | 193 |
| Male (n, %) | 9 (69) | 14 (64) | 137 (71) |
| Age (median, IQR) | 27 [24; 28] | 33 [30;37]. (n=9) | 33 [30; 39]. (n=177) |
| Time to diagnosis (months, median, IQR) | 9 [1;28] | 24 [24;27]. (n=7) | 24 [17; 51]. (n=130) |
| ISG set criteria positive (n, %) | 5 (38) | 17 (77) | 177 (92) |
| ICBD >= 4 | 12 (92) | 20 (91) | 190 (98) |
| Oral ulcers (n, %) | 10 (77) | 19 (86) | 192 (99) |
| Genital ulcers (n, %) | 7 (54) | 15 (68) | 162 (84) |
| Skin involvement (n, %) | 6 (46) | 15 (68) | 41(54) (n=76) |
| Vascular involvement (n, %) | 7 (54) | 7 (32) | 53 (27) |
| Ocular involvement (n, %) | 5 (38) | 14 (64) | 91 (47) |
| CNS involvement (n, %) | 3 (23) | 10 (45) | 68 (38) (n=180) |
| Positive allele HLA-B51 | 2 (100) (n=2) | 0 (0) (n=11) | 10 (16) (n=63) |

|  | **Egypt [59]** | **Turkey [60]** | **Europe [61]. (Spain)** | **Japan [62]** | **Sub-Saharan ancestry**  **[Review cohort]** |
| --- | --- | --- | --- | --- | --- |
| Number (n, %) | 1526 | 887 | 59 | 135 | 228 |
| Male (n, %) | 1102 (72) | 406 (46) | 29 (49) | 57 (42) | 160 (70) |
| Age, years, median [IQR]. or mean (SD) | 29 (9) | 38 (11) | 36 (12) | 38 (16) | 31 [26; 37]. (n=199) |
| Oral ulcers (n, %) | 1526 (100) | 887 (100) | 59 (100) | 124 (92) | 221 (97) |
| Genital ulcers (n, %) | 1297 (85) | 722 (83) | 46 (78) | 73 (54) | 184 (81) |
| Skin involvement (n, %) | 748 (49) | 682 (77) | 50 (85) | 102 (76) | 62 (56) (n=111) |
| Vascular involvement (n, %) | 366 (24) | 106 (12) | 6 (10) | 11 (8) | 67 (29) |
| Ocular involvement (n, %) | 1083 (71) | N/A | 55 (56) | 46 (34) | 110 (48) |
| CNS involvement (n, %) | 198 (13) | 34 (4) | 10 (17) | 17 (13) | 83 (38) (n=216) |
| Positive allele HLA-B51 | N/A | N/A | N/A | 28/72 (39) | 12 (16) (n=76) |

**Table S5:** Main characteristics of patients with Behçet’s disease according to ethnicity

N/A: data not available

Figure S1: Pooled frequencies of occurrence of fever of Behcet’s disease in adult patients of sub-Saharan ancestry

**e) Fever**


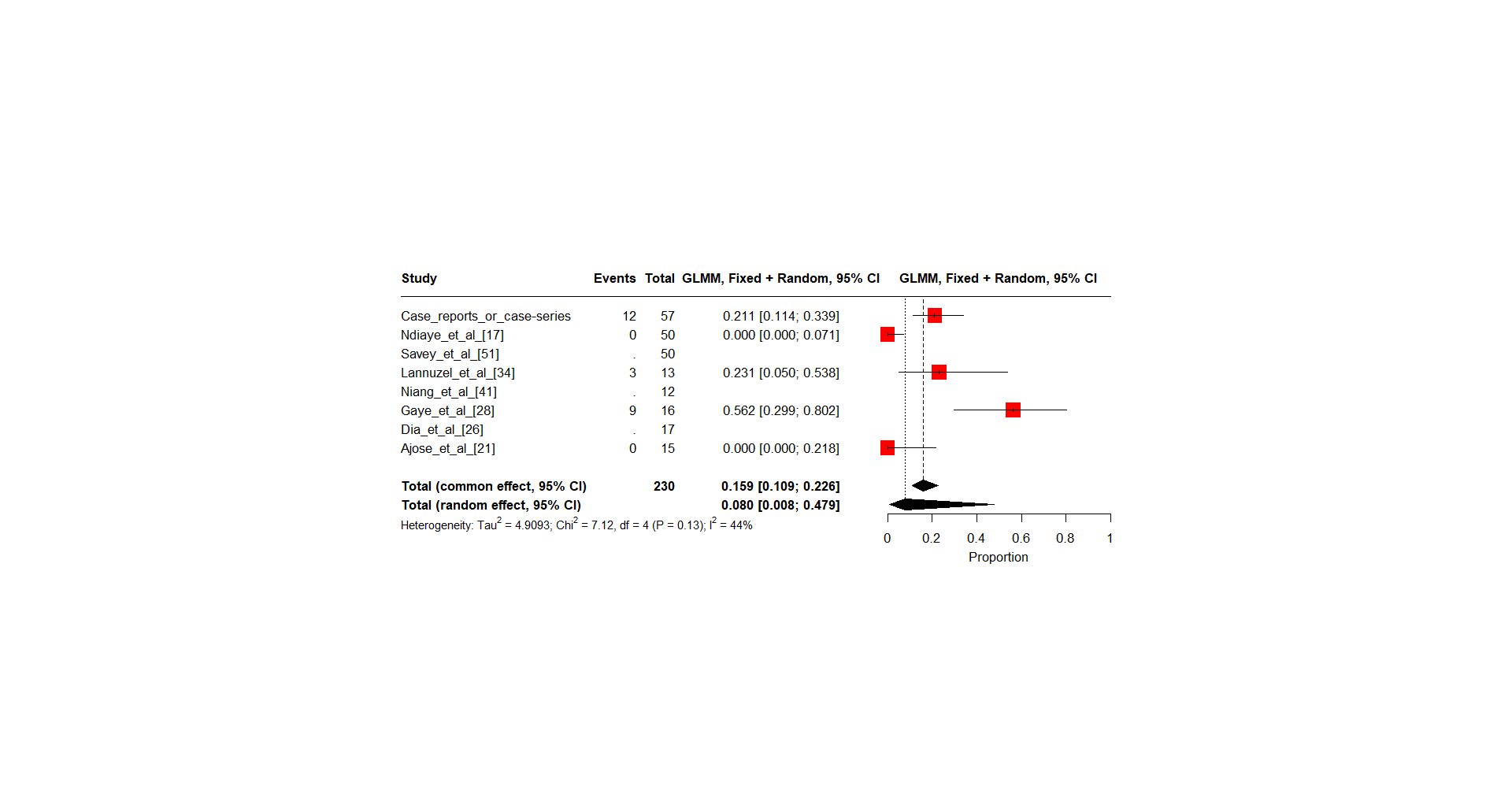


Figure S2 : pooled frequencies of male sex

**i) Male sex**


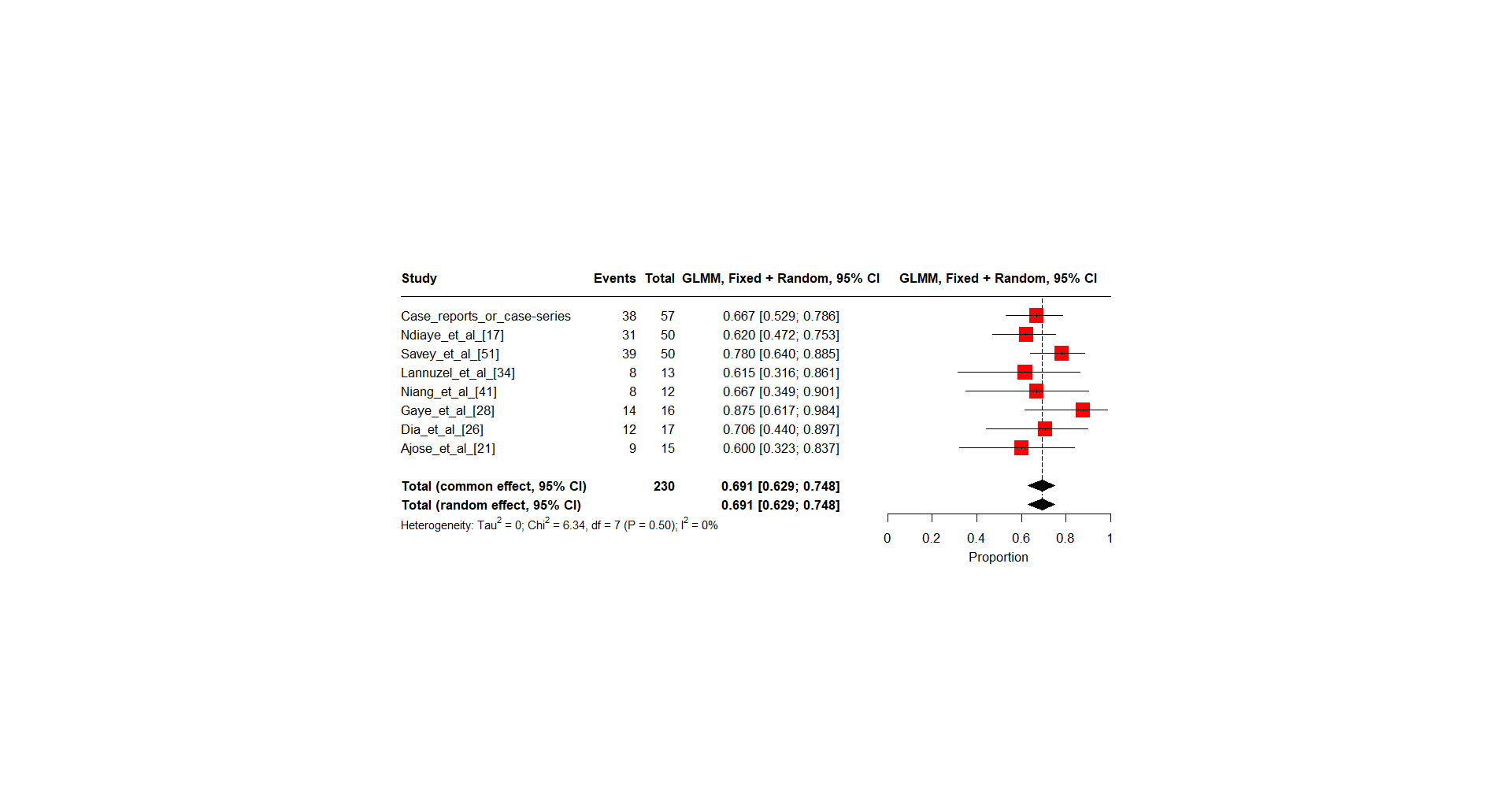

Supplement: Supplementary file 4 — Supplementary file4 (DOCX 161 KB) [file 12016_2025_9085_MOESM4_ESM.docx]
